# A Comprehensive Analysis of Key Immune Checkpoint Receptors on Tumor-Infiltrating T Cells From Multiple Types of Cancer

**DOI:** 10.3389/fonc.2019.01066

**Published:** 2019-10-25

**Authors:** Xi Li, Rouzheng Wang, Peiwen Fan, Xuan Yao, Ling Qin, Yanchun Peng, Miaomiao Ma, Neil Asley, Xuimei Chang, Yaning Feng, Yunhui Hu, Yonghong Zhang, Chris Li, Gregory Fanning, Stephanie Jones, Clare Verrill, David Maldonado-Perez, Paul Sopp, Craig Waugh, Stephen Taylor, Simon Mcgowan, Vincenzo Cerundolo, Christopher Conlon, Andrew McMichael, Shichun Lu, Xiyan Wang, Ning Li, Tao Dong

**Affiliations:** ^1^Key Laboratory of Tumor Immunology and Radiation Therapy, Third Affiliated Hospital, Xinjiang Tumor Hospital, Chinese Academy of Medical Sciences (CAMS), Xinjiang Medical University, Ürümqi, China; ^2^Nuffield Department of Medicine (NDM), Chinese Academy of Medical Sciences Oxford Institute (CAMS Oxford Institute), University of Oxford, Oxford, United Kingdom; ^3^MRC Human Immunology Unit, Radcliffe Department of Medicine, Weatherall Institute of Molecular Medicine, University of Oxford, Oxford, United Kingdom; ^4^Third Affiliated Hospital, Xinjiang Tumor Hospital, Xinjiang Medical University, Ürümqi, China; ^5^Beijing You'an Hospital, Capital Medical University, Beijing, China; ^6^Single Cell Genomics Facility, Weatherall Institute of Molecular Medicine, University of Oxford, Oxford, United Kingdom; ^7^China R&D, Janssen Pharmaceuticals, Shanghai, China; ^8^Oxford Radcliffe Biobank, Department of Cellular Pathology, Oxford University Hospitals NHS Trust, Oxford, United Kingdom; ^9^Nuffield Department of Surgical Sciences, NIHR Oxford Biomedical Research Centre, University of Oxford, Oxford, United Kingdom; ^10^Flow Cytometry Facility, Weatherall Institute of Molecular Medicine, University of Oxford, Oxford, United Kingdom; ^11^Bioinformatics Team, Weatherall Institute of Molecular Medicine, University of Oxford, Oxford, United Kingdom; ^12^China Military General Hospital, Beijing, China

**Keywords:** T cells, inhibitory receptor, tumor-infiltrating lymphocytes, tumor microenvironment, combinatorial checkpoint blockade

## Abstract

**Background:** Cancer patients often display dysfunctional antitumor T-cell responses. Because noteworthy benefits of immune checkpoint pathway blockade, such as programmed cell death protein 1 (PD-1) inhibitors, have been achieved in multiple advanced cancers, the next critical question is which mono-blockade or combinatorial blockade regimens may reinvigorate antitumor T-cell immunity in those cancer patients while limiting immune-related adverse effects.

**Method:** This study recruited, in total, 172 primary cancer patients (131 were blood-tumor-matched patients) who were treatment-naïve prior to the surgeries or biopsies covering the eight most prevalent types of cancer. With access to fresh surgical samples, this study simultaneously investigated the *ex vivo* expression level of eight known immune checkpoint receptors [PD-1, cytotoxic T-lymphocyte antigen-4 [CTLA-4], T-cell immunoglobulin and mucin-domain containing-3 [Tim-3], 2B4, killer cell lectin like receptor G1 [KLRG-1], TIGIT, B- and T-lymphocyte attenuator [BTLA], and CD160] on tumor-infiltrating T cells (TILs) and paired circulating T cells in blood from a 131-patient cohort.

**Results:** We found increased an expression of PD-1 and Tim-3 but a decreased expression of BTLA on TILs when compared with peripheral blood from multiple types of cancer. Moreover, our co-expression analysis of key immune checkpoint receptors delineates “shared” subsets as PD-1+Tim-3+TIGIT+2B4+KLRG-1–CTLA-4– and PD-1+TIGIT+2B4+Tim-3–KLRG-1–CTLA-4– from bulk CD8 TILs. Furthermore, we found that a higher frequency of advanced differentiation stage T cells (CD27–CCR7–CD45RA–) among the “shared” subset (PD-1+Tim-3+TIGIT+2B4+KLRG-1–CTLA-4–) in bulk CD8 TILs was associated with poorly differentiated cancer type in cervical cancer patients.

**Conclusions:** To our knowledge, our study is the first comprehensive analysis of key immune checkpoint receptors on T cells in treatment-naïve, primary cancer patients from the eight most prevalent types of cancer. These findings might provide useful information for future design of mono-blockade/combinatorial blockades and/or genetically modified T-cell immunotherapy.

## Introduction

The combinations of co-stimulatory and co-inhibitory receptors on T cells enable effective T-cell responses to viruses and tumors while also limiting immunopathology and autoimmunity (1, 2). However, this homeostasis can be disrupted by persistent antigen stimulation and immunosuppressive mediators released in the tumor microenvironment, leading T cells to a dysfunctional state, known as T-cell exhaustion, which fails to control viruses, or cancer (3–5). Intensive studies of dysfunctional T cells in cancers and in the context of chronic virus infection—for instance, with hepatitis B virus (HBV), hepatitis C virus (HCV), and human immunodeficiency virus (HIV)—suggested that exhausted T cells are characterized by an elevated expression of single or multiple inhibitory receptors (IRs) along with dysfunctional production of cytokines and activation of apoptotic signaling (6–10). However, the fundamental mechanism by which T cells become dysfunctional in the tumor microenvironment remains unclear.

In 2011, ipilimumab became the first approved blockade agent targeting the T-cell inhibitory receptor “cytotoxic T-lymphocyte antigen-4” (CTLA-4); advanced melanoma patients treated with ipilimumab demonstrated improved tumor control and survival (11). More recently, checkpoint blockade immunotherapy targeting “programmed cell death protein 1” (PD-1) has shown promise in treating multiple solid tumors, including metastatic melanoma, non-small-cell lung cancer (NSCLC), kidney cancer, urothelial cancer, head and neck cancer, and liver cancer (12–16). Consequently, the Federal Food and Drug Administration (FDA) in the USA accelerated the approval of clinical trials of PD-1 blockade in multiple cancers to benefit different types of advanced-cancer patients (15, 17, 18). Furthermore, PD-L1, as one of the ligands of PD-1, is capable of binding CD80 on activated T cells mediating inhibitory signaling (19, 20). Therefore, blocking PD-L1 with or without the co-blockade of PD-1 is a promising approach to restore the dysfunctional T cells in cancers (2). In fact, PD-L1 blockade has shown durable responses to induce tumor regression in patients with multiple cancers, with an overall response rate (ORR) between 6 and 17% (21).

However, PD-1 blocking agents such as pembrolizumab or nivolumab do not demonstrate antitumor effects in all cancer patients. For instance, pembrolizumab achieved a 19.4% ORR in treating advanced NSCLC, just over 20% ORR in treating advanced urothelial cancer, 22% ORR in treating advanced gastric cancer, and 18.5% ORR in treating advanced triple negative breast cancer (13, 14, 22–24). Additionally, adverse effects have been widely observed in patients treated with CTLA-4 or PD-1 blocking agents (25–27). In a PD-1 advanced melanoma clinical trial, drug-related adverse effects were reported in 79% of patients (107/135), and 13% of patients (17/135) demonstrated grade 3 or 4 drug-related adverse effects (28). A recent systematic meta-analysis revealed increased rates of hypothyroidism (odds ratio = 7.56, 95% confidence interval 4.53–12.61), pneumonitis (odds ratio = 5.37, 95% confidence interval 2.73–10.56), colitis (odds ratio = 2.88, 95% confidence interval 1.3–6.37), and hypophysitis (odds ratio = 3.38, 95% confidence interval 1.02–11.08) in cancer patients receiving anti-PD-1 treatments compared with standard treatments (29).

The discovery of additional immunoinhibitory receptors on T cells creates more options for mono-blockades or combinatorial blockades. T-cell immunoglobulin and mucin-domain containing-3 (Tim-3) was first discovered in 2002 as an IR on T cells (30). Galectin-9, phosphatidylserine (PtdSer), high mobility group protein B1 (HMGB1), and carcinoembryonic antigen cell adhesion molecule 1 (CEASAM-1) are current known ligands that can interact with Tim-3, inhibiting effector T-cell function (31–36). “Killer cell lectin like receptor G1” (KLRG-1) was known as an IR and a T-cell differentiation marker (37–39). As an IR, KLRG-1 can bind with e-cadherin, inhibiting T-cell function via Akt signaling (40, 41). KLRG-1 blockade is able to restore the phosphorylation of Akt signaling resulting in the enhancement of proliferative activity of dysfunctional CD8 T cells (42, 43). Both B- and T-lymphocyte attenuator (BTLA) and CD160 are able to impair antitumor T-cell responses by binding with herpes virus entry mediator (HVEM) (44–46). CD244 (2B4) can bind with CD48 and has dual roles as a stimulatory receptor and an IR (47, 48). The inhibitory function induced through T-cell immunoglobulin and ITIM (TIGIT) was quite similar to that of CTLA-4. The binding between CD226 and CD155 could deliver stimulatory signaling in T cells and NK cells (49). However, TIGIT competitively binds with CD155 with much higher affinity than with CD226, delivering inhibitory signaling to T cells and NK cells (50). Given the breadth of potential immunoinhibitory pathways available in the tumor microenvironment in addition to PD-1 and CTLA-4, the next critical questions to tackle include the following: (1) Which immunoinhibitory pathway blockade regimens should be combined to enhance and sustain antitumor responses and thus improve cancer patient outcomes? (2) Which inhibitory pathway blockade might elicit severe autoimmunity and thus should be carefully managed?

In this study, we evaluated the potential risks, benefits, and suitability of potential blockade immunotherapy for eight of the most prevalent solid tumors. Considering the potential alteration that radiotherapy or chemotherapy may bring to the expression of IRs on tumor-infiltrating T cells (TILs), all the patients recruited in our cohort were treatment-naïve, primary cancer individuals ranging from the eight most prevalent cancer types (51). This valuable cohort allows us to comprehensively evaluate the overall expression of key IRs on T cells in patients with multiple types of cancer, aiming to provide useful information for both clinicians who practice blockade immunotherapy on advanced-cancer patients and scientists who devote themselves to characterizing the mechanisms of a specific inhibitory pathway on T cells. We investigated the surface expression of eight IRs on T cells in peripheral blood and tumor-infiltrating lymphocytes (TILs). We propose that systemic infusion of blocking agents that target IRs expressed highly in peripheral blood, such as BTLA, might trigger autoimmunity in patients. We observed co-expression of PD-1, Tim-3, and TIGIT on cytotoxic (CD8) TILs in multiple types of cancer, which indicates the phenotype of CD8 TILs for future *in vitro* analysis of dysfunctional T cells. Furthermore, we found that a high frequency of Tim-3+ CD8 TILs tended to associate with poorly differentiated cervical cancer. These data suggest that cancer differentiation type, a well-established routine clinical test, represents a potential biomarker for the suitability of Tim-3 blockade immunotherapy.

## Materials and Methods

### Study Subjects and Ethical Statement

Fresh surgical samples with paired peripheral blood of primary cancer patients were collected in Beijing You'an Hospital, Capital Medical University, and Xinjiang Tumor Hospital, Xinjiang Medical University. Written informed consent was obtained from all cancer patients. All the patients were diagnosed and confirmed as primary cancer individuals who have not received any anticancer treatments beforehand. Fresh tumor samples were collected from either surgeries or biopsies. All methods were performed in accordance with the relevant guidelines and regulations, with ethical approval obtained from the Oxford Radcliffe Biobank (ORB) research tissue bank ethics committee (OCHRE reference 17/A006; REC reference 09/H0606/5+5), Oxford Tropical Research Ethics Committee (OxTREC reference 587-16), and the First Affiliated Hospital of Xinjiang Medical University Ethics Committee and Beijing You'an Hospital Ethics Committee.

### Isolation of Lymphocytes From Blood and Tumor Tissues

Peripheral blood mononuclear cells (PBMCs) were isolated from fresh heparinized blood by Ficoll-Hypaque density gradient centrifugation. Surgical tumor tissues were immediately transferred to tumor dissociation solution-containing (Miltenyi Biotec, catalog no. 130-095-929) C tube (Miltenyi Biotec, catalog no. 130-093-237). The tissues were then dissected into 1- to 3-mm pieces by sterile surgical scissor (Ethicon, USA). C tubes were placed on Octo-gentle dissociator (Miltenyi Biotec, catalog no. 130-095-937). Human tumor program-1 was performed for the dissociation followed by 20-min incubation on the gentle-mix rotator (Miltenyi Biotec, catalog no. 130-090-753) at 37°C, 5% CO_2_ incubator. A 70-nm cell strainer (Sigma-Aldrich, Dorset, UK) was then used to purify the intra-tumor or intra-tissue lymphocytes. Further, cells were washed twice in R10 and counted by trypan blue staining.

### Multichromatic Flow Cytometry Staining

From 2012 to 2014, eight-color panels were designed for an *ex vivo* phenotypic analysis. From 2014 onwards, with an upgraded flow cytometer, a 14-color panel was designed for the surface analysis of multiple IRs on T cells, which allowed us to investigate the co-expression of multiple IRs on TILs. Reviewing the results from the study in the first 2 years, when we upgraded the panel from 2014 onwards, we decided to exclude BTLA and CD160 from the updated panel owing to their low expressions on TILs and to add TIGIT and three T-cell differentiation markers (CD27, CCR7, and CD45RA) to the 14-color panel. Subsequently, we conducted a co-expression analysis in patients with multiple types of cancer. The details of panels and antibodies are listed in Supplementary Table 2. Cells derived from paired tumor and PBMC sample were each initially stained with LIVE/DEAD® Fixable Aqua Dead Cell Stain Kit (Thermo Fisher Scientific) for 20 min before surface staining with conjugated antibodies in fluorescence-activated cell sorting (FACS) washing buffer [phosphate-buffered saline [PBS] with 0.5 M of ethylenediaminetetraacetic acid [EDTA] and 7.5% bovine serum albumin [BSA] solution] for another 20 min and fixed with 1 × CellFix solution (BD Biosciences). Commercial conjugated antibodies used include CD3-Alexa Fluor 700 (344822, BioLegend), CD4-FITC (345768, BD Biosciences), CD8-APC-Cy7 (560179, BD Biosciences), CD160-PE-cy7 (341212, BioLegend), BTLA-APC (344510, BioLegend), PD-1-BV650 (564104, BD Biosciences), Tim3-BV421 (345008, BioLegend), TIGIT-PE (12-9500-42, eBioscience), 2B4-APC (329512, BioLegend), KLRG-1-BV605 (138419, BioLegend), CTLA-4-PE-Cy7 (349914, BioLegend), CD27-PERCP5.5 (356407, BioLegend), CCR7-BV711 (353227, BioLegend), and CD45RA-BV785 (304139, BioLegend). The antibody cocktails were tested in advance with or without the use of tumor dissociation solution to ensure proper function. Fluorescence minus one (FMO) controls were applied accordingly in order to properly position gates. In order to ensure the quality of FACS data, we ruled out any tumor samples in which the viable CD3+ TILs were lower than 10,000 cells. The full gating strategy of two 8-color FACS panels and 14-color FACS panel is shown in Supplementary Figures 4–8. The exemplary gates of IRs on T cells are shown in Figures 2A,B, 4A,B.

### Statistical Analysis

Mono-expression analysis and correlation analysis graphs were generated and analyzed in GraphPad v.7 software (Prism). A co-expression analysis was conducted in Simplified Presentation of Incredibly Complex Evaluations (SPICE) software [National Institute of Allergy and Infectious Diseases (NIAID)]. Unless stated otherwise, data are summarized as median ± s.e.m. Statistically significant differences between two groups were assessed using a two-tailed paired *t*-test, with Wilcoxon adjustments for non-parametrically distributed variables. Differences were considered statistically significant at *P* < 0.05, (^*^P < 0.0332, ^**^P < 0.0021, ^***^P < 0.0002, and ^****^P < 0.0001).

## Results

### Differential Surface Expression of Inhibitory Receptors on T Cells in Blood vs. Tumors From Cancer Patients

**Figure 1:**
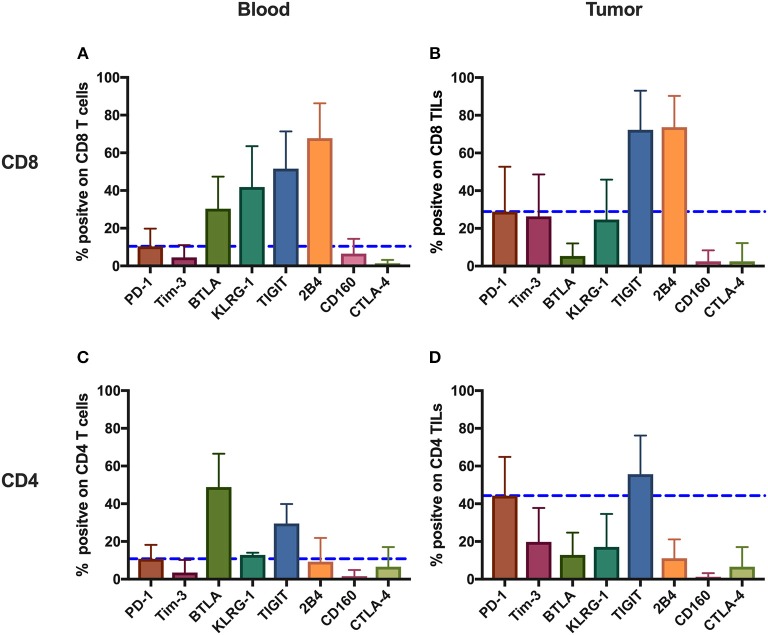
Inhibitory receptors have distinct frequencies in T-cell populations from peripheral blood and tumor tissues from eight types of cancer patients. FACS analysis reveals the frequency of PD-1+, Tim-3+, BTLA+, TIGIT+, 2B4+, CD160+, and CTLA-4+ cells in peripheral CD8 T cells **(A)**, CD8 TILs **(B)**, peripheral CD4 T cells **(C)**, and CD4 TILs **(D)**. T cells were obtained from 131 primary cancer patients, including 38 with breast cancer, 10 with liver cancer, 11 with lung cancer, 9 with esophageal cancer, 14 with gastric cancer, 15 with colorectal cancer, 14 with kidney cancer, and 20 with cervical cancer. The blue dotted line is the mean frequency of PD-1+ cells on each graph. The abovementioned IRs were investigated in three different panels (details are shown in Supplementary Table 2). 131 cancer patients were used for the analysis of PD-1, Tim-3, KLRG-1, and 2B4; 74 cancer patients were used for the analysis of BTLA and CD160; and 67 cancer patients were investigated for the analysis of TIGIT. Details of the overlapping patients and the number of the patients in each cancer cohort are listed in Supplementary Table 4. FACS, fluorescence-activated cell sorting; TILs, tumor-infiltrating lymphocytes; PD-1, programmed cell death protein 1; Tim-3, T-cell immunoglobulin and mucin-domain containing-3; KLRG-1, killer cell lectin like receptor G1; 2B4, CD244; BTLA, B- and T-lymphocyte attenuator.

To comprehensively investigate the immunoinhibitory pathways in T cells, we used FACS to analyze the surface expression of eight IRs (PD-1, Tim-3, BTLA, KLRG-1, TIGIT, 2B4, CD160, and CTLA-4) on T cells from peripheral blood and tumors isolated from 131 cancer patients (Supplementary Table 1, Figure 1). Having updated the panel in 2014, removing CD160 and BTLA, and adding TIGIT, the number of patients used for the expression analysis of BTLA and CD160 and that of TIGIT is different from that of other IRs. Details of the overlapping of patients for different IR analyses can be found in Supplementary Table 4. We observed distinct surface expression of IRs on CD8 (cytotoxic) T cells compared with CD4 T cells from peripheral blood. For instance, 2B4+ and KLRG-1+ cells displayed a high frequency in the CD8 circulating T-cell population but an almost five times lower frequency in the population of CD4 circulating T cells. In contrast, PD-1+ cells showed a similar and low frequency in circulating CD4 T cells and CD8 T cells. We also observed unique expression patterns in TILs. For instance, although KLRG-1+ cells displayed a relatively low frequency in both CD8 and CD4 TILs, 2B4+ cells accounted for almost 80% of CD8 TILs.

We also compared the mean frequencies of cells expressing IRs with the mean frequency of PD-1+ cells. Peripheral CD8 T cells showed two-fold higher frequencies of BTLA+, KLRG-1+, TIGIT+, and 2B4+ cells than did PD-1+. Further, CD8 TILs, relative to PD-1+, showed two-fold higher mean frequencies of TIGIT+ and 2B4+ cells. Circulating CD4 T cells, relative to PD-1+, showed higher frequencies of BTLA+ and TIGIT+, but only TIGIT+ CD4 TILs were more frequent than PD-1+ CD4 TILs.

Generally, the IRs were expressed more frequently on CD8 T cells compared with CD4 T cells and on TILs compared with circulating T cells, the exception being BTLA surface expression, which showed the highest frequency on circulating CD4 T cells from patients.

### Increased PD-1+ and Tim-3+ but Decreased BTLA+ T-Cell Frequencies Are Observed in Tumors When Compared With Peripheral Blood From Individual Patients Across Multiple Cancer Types

**Figure 2:**
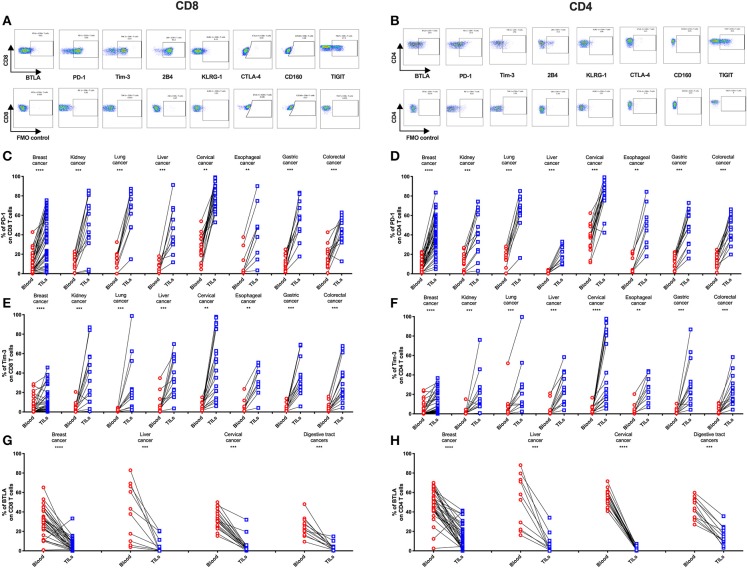
Higher frequencies of PD-1 and Tim-3 but lower frequencies of BTLA in T cells from tumors compared with peripheral blood in matched patient samples. Representative dot plots of eight inhibitory receptors on CD8 **(A)** and CD4 **(B)** TILs with FMO controls are shown. The exemplary plots are combined from two 8-color panels of two breast cancer patients. Full gating strategy of two 8-color panels and the 14-color panel is shown in Supplementary Figures 4–8. FACS analysis of PD-1 **(C,D)** and Tim-3 **(E,F)** in CD8 (left) and CD4 (right) T cells was performed for 131 blood-tumor-matched primary cancer patients (see Figure 1 for details). Analysis of BTLA+ cells in CD8 **(G)** and CD4 **(H)** T cells was performed for 74 blood-tumor-matched cancer patients: 32 breast cancer, 10 liver cancer, 20 cervical cancer, and 12 digestive tract cancer (five esophageal cancer, four gastric cancer, and three colorectal cancer). Wilcoxon paired *t*-test was performed to detect the statistical significance (^*^P < 0.0332, ^**^P < 0.0021, ^***^P < 0.0002, and ^****^P < 0.0001). The fraction of cells expressing PD-1, Tim-3, or BTLA on the surface of T cells in peripheral blood vs. tumor is shown as %. PD-1, programmed cell death protein 1; Tim-3, T-cell immunoglobulin, and mucin-domain containing-3; BTLA, B-, and T-lymphocyte attenuator; TILs, tumor-infiltrating lymphocytes; FMO, fluorescence minus one; FACS, fluorescence-activated cell sorting.

To determine if the IRs are differentially expressed on TILs vs. peripheral T cells from individual patients, we used FACS to analyze blood-tumor-matched samples from the 131 primary cancer patients (clinical characteristics are listed in Supplementary Table 1). We detected significantly higher frequencies of PD-1+ and Tim-3+ cells in TILs compared with their counterparts from peripheral blood across eight types of cancer (Figures 2C–F). In contrast, the frequency of BTLA+ cells significantly decreased in CD4 and CD8 TIL populations when compared with the respective peripheral blood populations across multiple cancer types from 74 of these cancer patients (Figures 2G,H, where esophageal, gastric, and colorectal cancers were analyzed together as “digestive tract cancers”). Further, we discovered that the frequencies of PD-1+ and Tim-3+ cells in peripheral T cells positively correlated with their respective frequencies in TILs (Supplementary Figures 1A–D). In contrast, BTLA surface expression in peripheral T cells and TILs did not correlate (Supplementary Figures 1E,F). Similarly, we observed an increased frequency of TIGIT+ cells in both CD4 and CD8 TILs when compared with T cells in peripheral blood from lung, cervical, gastric, and colorectal cancer patients; breast and esophageal cancer patients showed a similar trend, but it was not statistically significant (Supplementary Figures 2E,F). In contrast, the frequencies of 2B4+ (Supplementary Figures 2A,B) and CD160+ (Supplementary Figures 2I,J) T cells were similar in peripheral blood and tumor; the frequency of 2B4+ cells was high in both peripheral and tumor-infiltrating CD8 T cells (Supplementary Figure 2A).

KLRG-1+ CD8 T cells displayed a lower frequency in tumors compared with peripheral blood from patients with breast, cervical, esophageal, gastric, and colorectal cancers but not with kidney, lung, and liver cancers (Supplementary Figure 2C). Although KLRG-1+ CD4 T cells showed a higher frequency in tumors compared with blood from kidney cancer patients, they displayed similar frequencies in blood and tumors from patients with the remaining cancer types (Supplementary Figure 2D).

Notably, we found that cells expressing CTLA-4, the first approved checkpoint blockade target, displayed a generally low frequency (<20%) in both CD4 and CD8 T cell populations in the tumor and peripheral blood across eight cancer types. However, multiple cancers showed an increased frequency of CTLA-4+ CD4 T cells, but not CTLA-4+ CD8 T cells, in tumors compared with peripheral blood (Supplementary Figures 2G,H).

### Co-expression of Inhibitory Receptors Identifies Shared Subsets of CD8 TILs in Multiple Types of Cancer

**Figure 3:**
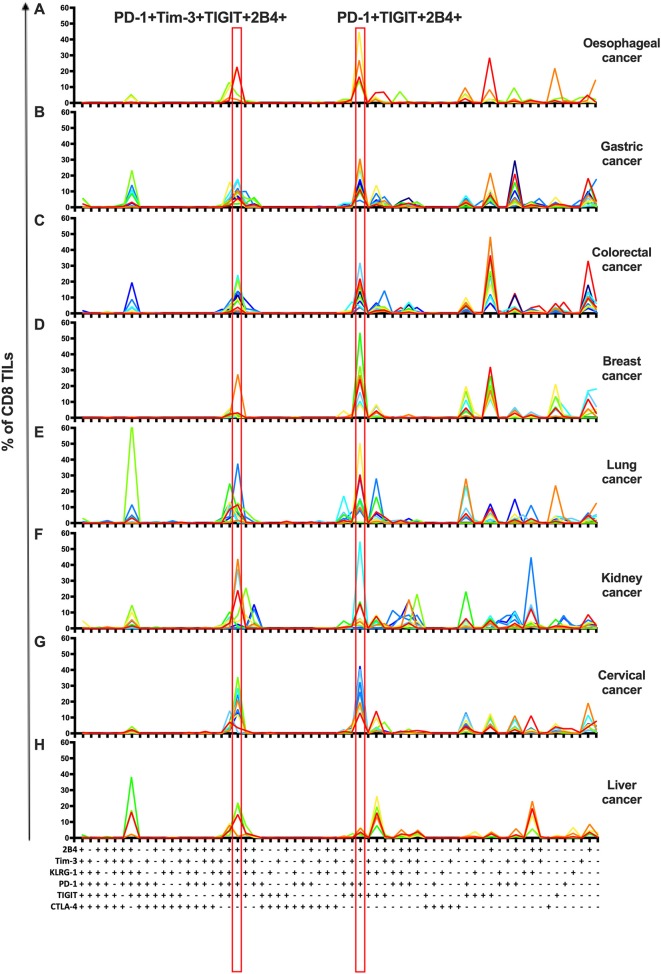
Co-expression analysis identifies shared subsets of CD8 TILs across multiple types of cancer. Simplified Presentation of Incredibly Complex Evaluations (SPICE) analysis was applied to investigate the co-expression of PD-1, Tim-3, 2B4, TIGIT, KLRG-1, and CTLA-4 on CD8 TILs from 69 cancer patients (4 with esophageal cancer, 11 with gastric cancer, 12 with colorectal cancer, 7 with breast cancer, 10 with lung cancer, 10 with kidney cancer, 10 with cervical cancer, and 5 with liver cancer). With six IRs investigated, there are 64 possible combinations/subsets (*X* axis) on CD8 TILs. Single color lines in each graph represent the frequency of different subsets of CD8 TILs from each patient, from eight types of cancer **(A–H)**. Red rectangles highlight the dominant subsets in CD8 TILs shared from 7/8 types of cancer. Co-expression analysis on CD8 TILs was conducted by 14-color panel, utilizing the full gating strategy shown in Supplementary Figures 8A,B. TILs, tumor-infiltrating lymphocytes; PD-1, programmed cell death protein 1; Tim-3, T-cell immunoglobulin, and mucin-domain containing-3; 2B4, CD244; TIGIT, T-cell immunoglobulin and ITIM; KLRG-1, killer cell lectin like receptor G1; CTLA-4, cytotoxic T-lymphocyte antigen-4; IR, inhibitory receptor.

To investigate the co-expression patterns of multiple IRs on CD8 TILs, we focused on PD-1, Tim-3, TIGIT, 2B4, KLRG-1, and CTLA-4. We identified PD-1+TIGIT+2B4+Tim-3+KLRG-1–CTLA-4– and PD-1+TIGIT+2B4+Tim-3–KLRG-1–CTLA-4– as dominant shared subsets of CD8 TILs in 64 cancer patients (4 with esophageal cancer, 11 with gastric cancer, 12 with colorectal cancer, 7 with breast cancer, 10 with lung cancer, 10 with kidney cancer, and 10 with cervical cancer) (Figures 3A–G). Of note, five liver cancer patients showed a distinct shared subset of 2B4+TIGIT+KLRG-1+1PD-1–Tim-3–CTLA-4– CD8 TILs (Figure 3H). Our co-expression analysis appears to underscore the prominent role of the PD-1, TIGIT, and Tim-3 IRs on CD8 TILs in T-cell exhaustion for multiple types of cancer.

### Tim-3 and TIGIT Are Preferentially Expressed on PD-1+ CD8 TILs in Cancer Patients

**Figure 4:**
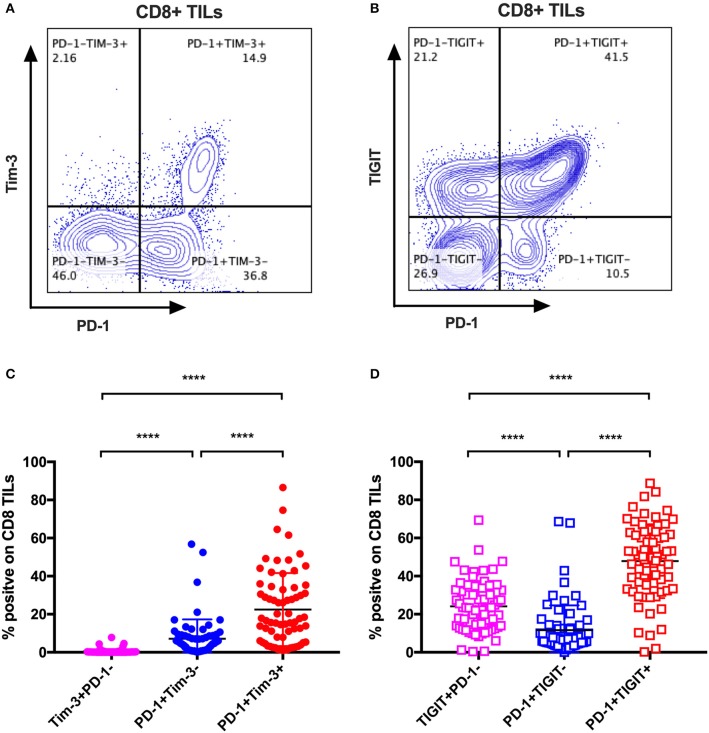
Tim-3 and TIGIT are preferentially expressed on PD-1+ CD8 TILs across multiple types of cancer. Representative dot plots of the co-expression of PD-1 and Tim-3 on CD8 TILs **(A)** and the co-expression of PD-1 and TIGIT on CD8 TILs **(B)** from a breast cancer patient are shown. Data were collected utilizing the 14-color panel as mentioned above, with full gating strategy shown in Supplementary Figure 8. **(C)** FACS analysis of the frequencies of Tim-3+PD-1–, PD-1+Tim-3–, and PD-1+Tim-3+ cells among CD8 TILs from 69 cancer patients (see Figure 3 for details). **(D)** FACS analysis of the frequencies of TIGIT+PD-1–, PD-1+TIGIT–, and PD-1+TIGIT+ cells among CD8 TILs from the same 69 cancer patients. Wilcoxon paired *t*-test was performed to detect the statistical significance (^*^P < 0.0332, ^**^P < 0.0021, ^***^P < 0.0002, and ^****^P < 0.0001). Tim-3, T-cell immunoglobulin and mucin-domain containing-3; TIGIT, T-cell immunoglobulin and ITIM; TILs, tumor-infiltrating lymphocytes; PD-1, programmed cell death protein 1; FACS, fluorescence-activated cell sorting.

The mono-expression and combinatorial expression analyses suggested the importance of PD-1, Tim-3, and TIGIT on CD8 T cells. We specifically investigated the co-expression of Tim-3 with PD-1 on CD8 TILs from the same cohort of cancer patients as mentioned in the co-expression analysis of IRs on CD8 TILs. We found that Tim-3+PD-1– CD8 TILs accounted for <5% of bulk CD8 TILs (Figure 4C), whereas PD-1+Tim-3– and PD-1+Tim-3+ CD8 TILs accounted for about 10 and around 20%, respectively, of CD8 TILs (Figure 4C). We then investigated the co-expression of TIGIT and PD-1 on CD8 TILs from the same cohort. Approximately 50% of CD8 TILs were PD-1+TIGIT+, around 20% were TIGIT+PD-1–, and about 10% were PD-1+TIGIT– (Figure 4D). Thus, Tim-3 and TIGIT are preferentially expressed on PD-1+ CD8 TILs.

### High Tim-3+ CD8 TILs Frequency Associates With Cervical Cancer Differentiation Status but Not Stage

**Figure 5:**
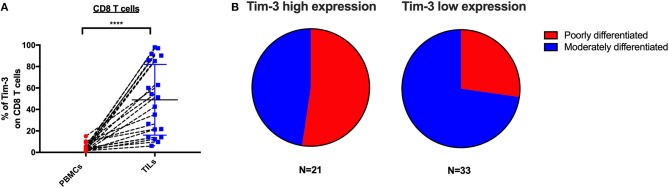
Tim-3 expression on CD8 TILs is associated with poorly differentiated cervical cancer. Association analysis between Tim-3 surface expression on T cells and differentiation and stage of cervical cancer patients. **(A)** Frequency of Tim-3 on CD4 and CD8 T cells in 20 blood-tumor tissues-matched samples from cervical cancer patients. **(B)** Fraction of poorly differentiated and moderately differentiated samples from the Tim-3 high-frequency subgroup and the Tim-3 low-frequency subgroup in 54 cervical cancer patients (20 blood-tumor tissue-matched patients and 34 tumor tissue-only patients). Mean frequency of Tim-3 was utilized to define Tim-3 high- and low-frequency subgroups. In the blood-tumor tissue-matched analysis, Wilcoxon paired *t*-test was performed to detect the statistical significance (^*^P < 0.0332, ^**^P < 0.0021, ^***^P < 0.0002, and ^****^P < 0.0001). Tim-3, T-cell immunoglobulin and mucin-domain containing-3; TILs, tumor-infiltrating lymphocytes.

To determine if the expression of IRs on CD8 TILs associates with any clinical characteristics, we investigated 54 cervical cancer patients of whom 20 are blood-tumor tissue-matched patients and 34 are tumor tissue-only patients in our clinical cohort. In the expression analysis of Tim-3 on CD8 TILs from 20 blood-tumor tissue-matched cervical cancer patients, we detected two sub-clusters, a Tim-3 high-frequency group and a Tim-3 low-frequency group (Figure 5A). We used the mean frequency of Tim-3 as a cutoff value to classify all cervical cancer patients into two subgroups. Based on the pathological diagnosis, in 54 cervical tumor tissues, the Tim-3 high-frequency subgroup had a higher percentage (52%, 11/21) of poorly differentiated cancer types than had the Tim-3 low-frequency subgroup (27%, 9/33) in cervical cancer patients, although we did not detect statistical significance of Tim-3+ CD8 TIL frequency between poorly differentiated and moderately differentiated cervical cancer patients (Figure 5B, Supplementary Figure 3A). When stratifying all the cervical cancer patients by their cancer stage, we did not observe a differential frequency of Tim-3+ CD8 TILs (Supplementary Figure 3B).

### Advanced Differentiation of the Shared PD-1+Tim-3+TIGIT+2B4+KLRG-1–CTLA-4– CD8 TILs Subset Is Associated With Poorly Differentiated Cervical Cancer

**Figure 6:**
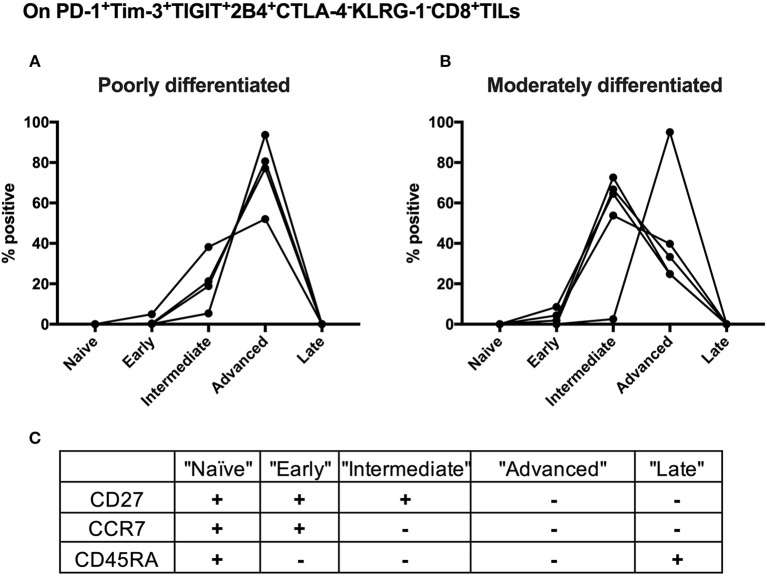
Advanced T cell-differentiated subset in the “shared” subset of CD8 TILs is linked to poorly differentiated cervical cancer. Having gated on PD-1+Tim-3+TIGIT+2B4+CTLA-4–KLRG-1–CD8+ TILs from 10 cervical cancer patients, Boolean combination gating strategy was then used to determine different T-cell differentiation stages by the surface expression of CD27, CCR7, and CD45RA. The frequency of each stage of TILs in the “shared” subset of CD8 TILs was depicted between five poorly differentiated cervical cancer samples **(A)** and five moderately differentiated cervical cancer samples **(B)**. Five T-cell differentiation stages **(C)** are “naïve” T cells (CD27+CCR7+CD45RA+), “early” differentiated T cells (CD27+CCR7+CD45RA–), “intermediate” differentiated T cells (CD27+CCR7–CD45RA–), “advanced” differentiated T cells (CD27–CCR7–CD45RA–), and “late” differentiated T cells (CD27–CCR7–CD45RA+). Representative dot plots of CD45RA, CD27, and C-C chemokine receptor type 7 (CCR7) on CD8 TILs with FMO controls are shown in Supplementary Figure 8B. The frequency of five different T-cell differentiation stages was then calculated by the function of Boolean Combination Gates in Flowjo software. TILs, tumor-infiltrating lymphocytes; FMO, fluorescence minus one.

We further conducted T-cell differentiation analysis of the identified shared subset, PD-1+TIGIT+2B4+Tim-3+KLRG-1–CTLA-4– in bulk CD8 TILs from 10 cervical cancer patients. We stratified 10 cervical cancer patients into two subgroups by pathology (five poorly differentiated and five moderately differentiated tumors). CD8 T-cell differentiation stages are assessed by the differential CD27, CCR7, and CD45RA expression (Figure 6C). We found that the majority (over 60%) of PD-1+TIGIT+2B4+Tim-3+KLRG-1–CTLA-4– CD8 TILs from all (5/5) of the poorly differentiated cervical cancer samples displayed an advanced T-cell differentiation phenotype and that only a small fraction (20%) displayed intermediate T-cell differentiation phenotypes (Figure 6A). Conversely, the majority (60%) of PD-1+TIGIT+2B4+Tim-3+KLRG-1–CTLA-4– CD8 TILs from most (4/5) of the moderately differentiated cervical cancer samples displayed an intermediate T-cell differentiation phenotype, and a minor fraction (40%) displayed advanced T-cell differentiation phenotypes (Figure 6B). These results suggest that the advanced T-cell differentiation (CD27–CCR7–CD45RA–) of PD-1+TIGIT+2B4+Tim-3+KLRG-1–CTLA-4– CD8 TILs is associated with poorly differentiated cervical cancer.

## Discussion and Conclusions

We have comprehensively investigated the surface expression of eight IRs on T cells from a valuable clinical cohort comprising only recruited primary cancer, treatment-naïve patients from China and the UK. We demonstrated distinct surface expression of IRs on T cells between peripheral blood and tumor tissues across multiple cancer types, and across CD4 vs. CD8 T cells. PD-1+ and Tim-3+ T cells were increased in tumors compared with peripheral blood across eight cancer types. These results are in line with previous studies of breast cancer, esophageal cancer, lung cancer, and liver cancer (52–55). Our study also found that BTLA+ T cells were decreased in tumors vs. blood, which is consistent with a recent breast cancer study (52). However, it has been reported in a previous melanoma study that the tumor microenvironment upregulates the expression of BTLA on CD8 TILs and that the co-expression of BTLA and PD-1 marks tumor-specific TILs in melanoma patients (56). In addition, we did not observe any differential expression of any individual IR on TILs in patients among different clinical stages in each type of cancer (data not shown). This is possibly due to the limited number of patients in each stage of an individual cancer type.

Releasing the brakes of IRs can be a double-edged sword: restoring T-cell antitumor responses but inducing immunopathology and breaking self-tolerance (57, 58). Patients in clinical trials of nivolumab and pembrolizumab to treat multiple types of cancer showed significantly higher risk of pneumonitis, colitis, hypothyroidism, hypophysitis, and hepatitis (15, 27, 59). These findings raise concerns about autoimmunity and immunopathology during the development of new blockade immunotherapies. On the basis of our results, we would categorize IRs into four groups, which are summarized in Table 1: group 1 IRs show a lower frequency on T cells in the circulation vs. tumors, such as PD-1 and Tim-3. The immune-related adverse effects generated by blocking these inhibition pathways are predicted to be manageable. Group 2 IRs show a higher frequency on T cells in the circulation vs. tumors, such as BTLA and KLRG-1. BTLA is linked with T-cell differentiation and is predominately expressed on naïve T cells (60). A recent functional study of KLRG-1 in animal models also revealed that after receiving stimulatory and inflammatory signals, KLRG-1+ CD8 T cells downregulated KLRG-1 and differentiated into different memory T-cell linages, with distinctive antivirus and antitumor capacities (61). Group 2 IRs may have less value in checkpoint blockade owing to the likelihood of immune-related adverse effects resulting from their relatively high expression on peripheral T cells. However, they may be suitable for genetic manipulation of antigen-specific T cells, which might limit the immune-related adverse effects caused by non-tumor-specific T cells. Furthermore, local injection of the group 2 IR-blocking agents into the tumor may reduce the immune-related adverse effects when compared to those in systemic infusion. Group 3 IRs show a high frequency on T cells in both peripheral blood and tumors, such as TIGIT and 2B4. Blocking these inhibition pathways might cause severe immune-related adverse effects, given their expression in peripheral blood T cells. Similarly to BTLA, these may not be ideal targets for checkpoint blockade that is administered via systemic infusion to patients. However, local injection rather than systemic infusion of blocking agents targeting the TIGIT or 2B4 pathways might minimize the immune-related adverse effects but still benefit patients. Of note, a previous study has shown that 2B4 could act as the ligand rather than receptor on target cells, which interact with CD48 expressing NK cells, activating human NK cells' cytotoxic functions (62). In chronic HCV and HBV infection, it was found that 2B4 was co-expressed with PD-1 on virus-specific CD8 T cells eliciting dysfunctional cytotoxic functions (6, 63). TIGIT has been revealed to be upregulated on peripheral T cells in elderly people, compared with young people, marking exhausted or senescent T cells in aging-related immunosuppression (64). Therefore, our interpretation is that the high expression level of TIGIT observed in patients from our cohort additively resulted from the presence of cancer and the average ages of patients (56.4 years old, details shown in Supplementary Table 1). Group 4 IRs show a low frequency on T cells from both peripheral blood and tumors, such as CTLA-4 and CD160. These results are consistent with previous reports that CTLA-4 and CD160 have soluble forms circulating in the blood (65, 66). As such, surface staining alone may not identify all CTLA-4+ or CD160+ T cells. In a lung cancer study, a higher frequency of CTLA-4 was found by intracellular staining in CD4 T cells when compared with those of surface staining in CD4 T cells from the same patients (67). Therefore, it is advisable to intracellularly stain CTLA-4 in T cells. It is also true that we are not sure about the expression of CTLA-4 on T cells in lymphatic organs such as the spleen or lymph nodes in various locations where T cells are primed. Furthermore, our study only measured the frequency of CTLA-4 on bulk CD4 TILs, but not in different CD4 subsets in the tumor. It was reported that Tregs in the tumor have higher expression of CTLA-4 than have conventional CD4 TILs (8). More importantly, numerous CTLA-4 blockade animal experiments and human clinical trials have suggested that blocking CTLA-4 can lead to high-grade immune-related adverse effects, which are of a more severe magnitude, compared with those of PD-1/PD-L1 blockade (68). Therefore, even though high expression of CTLA-4 was not observed on TILs, the vigor of T-cell responses resulting from CTLA-4 blockade may still elicit potent antitumor responses against cancers.

**Table 1:** Summary of four categories of key inhibitory receptors, its expression features, autoimmunity concerns, and clinical implication.

**Group of IRs**	**Group 1**	**Group 2**	**Group 3**	**Group 4**
IR	PD-1, Tim-3	BTLA, KLRG-1	2B4, TIGIT	CTLA-4, CD160
Expression in peripheral blood in cancer patients	+	+++	+++	Express at a negligible level on T cells owing to soluble forms
Expression in tumor tissues in cancer patients	+++	+	+++	Express at a negligible level on T cells owing to soluble forms
Concerns of autoimmunity	Manageable	Probably capable of inducing strong immune-related adverse effects	Probably capable of inducing strong immune-related adverse effects	CTLA-4 induces strong immune-related adverse effects. The potential immune-related adverse effects of CD160 blockade are unknown
Implication for blockade drug administration	Systemic IV infusion or local injection	Local injection	Local injection	CTLA-4 has been administered via systemic infusion to patients
Other applications	Genetic manipulation of tumor associated antigen (TAA) specific T cells	Genetic manipulation of TAA-specific T cells	Genetic manipulation of TAA-specific T cells	Neutralizing antibodies targeting soluble forms

The co-expression of IRs on CD8 TILs in individual cancer patients may inform the design of effective combinatorial blockade regimens. Our co-expression analysis of IRs delineates the shared subset of bulk CD8 TILs as PD-1+TIGIT+2B4+Tim-3+KLRG-1–CTLA-4–, supporting the rationale of combinatorial blockade against PD-1, Tim-3, and TIGIT inhibitory pathways. Of note, our interpretation is that in the combinatorial blockade of PD-1, Tim-3, and TIGIT, it is advisable to administer the blocking agents of TIGIT by local injection but not systemic infusion in order to limit the potential immune-related adverse effects. TIGIT has been reported as a co-regulator of the expansion and function of tumor-specific T cells in advanced melanoma patients, suggesting that co-blockade of PD-1 and TIGIT might elicit potent antitumor responses (69). In 2018, a phase II clinical trial of anti-TIGIT combined with or without PD-L1 blockade in advanced NSCLC patients without epithelial growth factor receptor (EGFR) mutation or anaplastic lymphoma kinase (ALK) translocation was approved (NCT03563716). Further, Tim-3 blockade reinvigorated antitumor T-cell responses in lung cancer patients who developed adaptive resistance to PD-1 blockade (70). From 2015 onwards, the safety and toxicity of anti-Tim-3 agents such as TSR-022, Sym023, and MBG453 were evaluated in several phase I clinical trials in multiple types of cancer (NCT02817633, NCT03489343, and NCT02608268) (71). Recently, the combinatorial blockade of PD-1 and Tim-3 in a phase II clinical in liver cancer and multiple solid tumors was initiated in 2018 (NCT03680508 and NCT03744468).

Both our data and a lung cancer study found that Tim-3 and TIGIT are preferentially expressed on PD-1+ CD8 TILs in multiple types of cancer, suggesting that combined blockade of these pathways could be effective to treat diverse tumors (72). One liver cancer study indicated that co-blockade of PD-1, CTLA-4, and Tim-3 pathways additively restores the functionality of exhausted CD8 TILs against cancer (54). Another hepatocellular carcinoma (HCC) investigation showed that combinatorial blockade of PD-1 and Tim-3 significantly enhanced the proliferative capacity of CD8 TILs from PD-1 high-expressers (73).

We found that Tim-3+ CD8 TILs might associate with poor cervical cancer differentiation but not stage. Our data further suggest that advanced T-cell differentiation of the shared subset from CD8 TILs in cervical cancer patients also associates with poorly differentiated cervical cancer. In clinical practice, poorly differentiated cancer correlates with poor prognosis. Our findings suggest that cancer differentiation status, which is routinely tested, might be a biomarker to identify cervical cancer patients who would respond to Tim-3 blockade.

To our knowledge, our study represents the first comprehensive analysis of IRs on T cells in blood and tumors from diverse cancer patients. Our findings illuminate the potential risks and benefits of future checkpoint blockades, or of genetic modified T-cell immunotherapy.

## Data Availability Statement

All data generated or analyzed during this study are included in this published article (and its supplementary information files).

## Ethics Statement

All procedures involving human subjects, tissues, and clinical data have been performed in accordance with the Declaration of Helsinki. All methods were performed in accordance to the relevant guidelines and regulations, with ethical approval obtained from the Oxford Radcliffe Biobank (ORB) research tissue bank ethics (OCHRE reference 17/A006; REC reference 09/H0606/5+5), Oxford Tropical Research Ethics Committee (OxTREC reference 587-16), and the First Affiliated Hospital of Xinjiang Medical University Ethics Committee and Beijing You'an Hospital Ethics Committee. Written informed consent has been signed by all the participants.

## Author Contributions

TD, XL, RW, and NL: study design. XL, XY, PF, MM, CC, YF, LQ, YH, YP, SL, ST, NA, SM, SJ, CV, DM-P, PS, CW, and YZ: data acquisition. XL, PS, NA, SM, NL, CL, and XW: data analysis. XL, TD, VC, CC, and AM: paper writing and edition. All authors read and approved the final manuscript.

### Conflict of Interest

The authors declare that the research was conducted in the absence of any commercial or financial relationships that could be construed as a potential conflict of interest.

## Abbreviations

2B4: CD244
ALK: Anaplastic lymphoma kinase
BRC: Biomedical Research Center
BSA: Bovine serum albumin
BTLA: B- and T-lymphocyte attenuator
CAMS: Chinese Academy of Medical Sciences
CCR7: C-C chemokine receptor type 7
CTLA-4: Cytotoxic T-lymphocyte antigen-4
EDTA: Ethylenediaminetetraacetic acid
EGFR: Epithelial growth factor receptor
FACS: Fluorescence-activated cell sorting
FDA: Federal Food and Drug Administration
FMO: Fluorescence minus one
HBV: Hepatitis B virus
HCC: Hepatocellular carcinoma
HCV: Hepatitis C virus
HIV: Human immunodeficiency virus
IR: Inhibitory receptor
KLRG-1: Killer cell lectin like receptor G1
NDM: Nuffield Department of Medicine
NIAID: National Institute of Allergy and Infectious Diseases
NIHR: National Institute for Health Research
NSCLC: Non-small-cell lung cancer
ORB: Oxford Radcliffe Biobank
ORR: Overall response rate
OxTREC: Oxford Tropical Research Ethics Committee
PBMC: Peripheral blood mononuclear cell
PBS: Phosphate-buffered saline
PD-1: Programmed cell death protein 1
SPICE: Simplified Presentation of Incredibly Complex Evaluations
TAA: Tumor-associated antigen
TCR: T-cell receptor
TIGIT: T-cell immunoglobulin and ITIM
TILs: Tumor-infiltrating lymphocytes
Tim-3: T-cell immunoglobulin and mucin-domain containing-3.

## References

[1] Maini MK Schurich A. The molecular basis of the failed immune response in chronic HBV: therapeutic implications. J Hepatol. (2010) 52:616–9. 10.1016/j.jhep.2009.12.017 20185199

[2] Fife BT Pauken KE Eagar TN Obu T Wu J Tang Q. Interactions between PD-1 and PD-L1 promote tolerance by blocking the TCR-induced stop signal. Nat Immunol. (2009) 10:1185–92. 10.1038/ni.1790 19783989 PMC2778301

[3] Pauken KE Wherry EJ. Overcoming T cell exhaustion in infection and cancer. Trends Immunol. (2015) 36:265–76. 10.1016/j.it.2015.02.008 25797516 PMC4393798

[4] Crespo J Sun H Welling TH Tian Z Zou W. T cell anergy, exhaustion, senescence, and stemness in the tumor microenvironment. Curr Opin Immunol. (2013) 25:214–21. 10.1016/j.coi.2012.12.003 23298609 PMC3636159

[5] Jiang Y Li Y Zhu B. T-cell exhaustion in the tumor microenvironment. Cell Death Dis. (2015) 6:e1792. 10.1038/cddis.2015.162 26086965 PMC4669840

[6] Bengsch B Martin B Thimme R. Restoration of HBV-specific CD8+ T cell function by PD-1 blockade in inactive carrier patients is linked to T cell differentiation. J Hepatol. (2014) 61:1212–9. 10.1016/j.jhep.2014.07.005 25016223

[7] Jones RB Ndhlovu LC Barbour JD Sheth PM Jha AR Long BR. Tim-3 expression defines a novel population of dysfunctional T cells with highly elevated frequencies in progressive HIV-1 infection. J Exp Med. (2008) 205:2763–79. 10.1084/jem.20081398 19001139 PMC2585847

[8] Montler R Bell RB Thalhofer C Leidner R Feng Z Fox BA. OX40, PD-1 and CTLA-4 are selectively expressed on tumor-infiltrating T cells in head and neck cancer. Clin Transl Immunol. (2016) 5:e70. 10.1038/cti.2016.16 27195113 PMC4855266

[9] Singer M Wang C Cong L Marjanovic ND Kowalczyk MS Zhang H. A distinct gene module for dysfunction uncoupled from activation in tumor-infiltrating T cells. Cell. (2016) 166:1500–11 e9. 10.1016/j.cell.2016.08.052 27610572 PMC5019125

[10] Schietinger A Philip M Krisnawan VE Chiu EY Delrow JJ Basom RS. Tumor-specific T cell dysfunction is a dynamic antigen-driven differentiation program initiated early during tumorigenesis. Immunity. (2016) 45:389–401. 10.1016/j.immuni.2016.07.011 27521269 PMC5119632

[11] Lipson EJ Drake CG. Ipilimumab: an anti-CTLA-4 antibody for metastatic melanoma. Clin Cancer Res. (2011) 17:6958–62. 10.1158/1078-0432.CCR-11-1595 21900389 PMC3575079

[12] Prieto PA Yang JC Sherry RM Hughes MS Kammula US White DE. CTLA-4 blockade with ipilimumab: long-term follow-up of 177 patients with metastatic melanoma. Clin Cancer Res. (2012) 18:2039–47. 10.1158/1078-0432.CCR-11-1823 22271879 PMC3319861

[13] Bellmunt J de Wit R Vaughn DJ Fradet Y Lee JL Fong L. Pembrolizumab as second-line therapy for advanced urothelial carcinoma. N Engl J Med. (2017) 376:1015–26. 10.1056/NEJMoa1613683 28212060 PMC5635424

[14] Garon EB Rizvi NA Hui R Leighl N Balmanoukian AS Eder JP. Pembrolizumab for the treatment of non-small-cell lung cancer. N Engl J Med. (2015) 372:2018–28. 10.1056/NEJMoa1501824 25891174

[15] Sharma P Retz M Siefker-Radtke A Baron A Necchi A Bedke J. Nivolumab in metastatic urothelial carcinoma after platinum therapy (CheckMate 275): a multicentre, single-arm, phase 2 trial. Lancet Oncol. (2017) 18:312–22. 10.1016/S1470-2045(17)30065-7 28131785

[16] Sorscher S. Pembrolizumab in non-small-cell lung cancer. N Engl J Med. (2017) 376:996–7. 10.1056/NEJMc1615559 28276230

[17] Chuk MK Chang JT Theoret MR Sampene E He K Weis SL. FDA approval summary: accelerated approval of pembrolizumab for second-line treatment of metastatic melanoma. Clin Cancer Res. (2017) 23:5666–70. 10.1158/1078-0432.CCR-16-0663 28235882

[18] Shu CA Rizvi NA. Into the clinic with nivolumab and pembrolizumab. Oncologist. (2016) 21:527–8. 10.1634/theoncologist.2016-0099 27026678 PMC4861376

[19] Park JJ Omiya R Matsumura Y Sakoda Y Kuramasu A Augustine MM. B7-H1/CD80 interaction is required for the induction and maintenance of peripheral T-cell tolerance. Blood. (2010) 116:1291–8. 10.1182/blood-2010-01-265975 20472828 PMC2938239

[20] Butte MJ Keir ME Phamduy TB Sharpe AH Freeman GJ. Programmed death-1 ligand 1 interacts specifically with the B7-1 costimulatory molecule to inhibit T cell responses. Immunity. (2007) 27:111–22. 10.1016/j.immuni.2007.05.016 17629517 PMC2707944

[21] Brahmer JR Tykodi SS Chow LQ Hwu WJ Topalian SL Hwu P. Safety and activity of anti-PD-L1 antibody in patients with advanced cancer. N Engl J Med. (2012) 366:2455–65. 10.1056/NEJMoa1200694 22658128 PMC3563263

[22] Muro K Chung HC Shankaran V Geva R Catenacci D Gupta S. Pembrolizumab for patients with PD-L1-positive advanced gastric cancer (KEYNOTE-012): a multicentre, open-label, phase 1b trial. Lancet Oncol. (2016) 17:717–26. 10.1016/S1470-2045(16)00175-3 27157491

[23] Nanda R Chow LQ Dees EC Berger R Gupta S Geva R. Pembrolizumab in patients with advanced triple-negative breast cancer: phase Ib KEYNOTE-012 study. J Clin Oncol. (2016) 34:2460–7. 10.1200/JCO.2015.64.8931 27138582 PMC6816000

[24] Plimack ER Bellmunt J Gupta S Berger R Chow LQ Juco J. Safety and activity of pembrolizumab in patients with locally advanced or metastatic urothelial cancer (KEYNOTE-012): a non-randomised, open-label, phase 1b study. Lancet Oncol. (2017) 18:212–20. 10.1016/S1470-2045(17)30007-4 28081914

[25] Brahmer JR Drake CG Wollner I Powderly JD Picus J Sharfman WH. Phase I study of single-agent anti-programmed death-1 (MDX-1106) in refractory solid tumors: safety, clinical activity, pharmacodynamics, and immunologic correlates. J Clin Oncol. (2010) 28:3167–75. 10.1200/JCO.2009.26.7609 20516446 PMC4834717

[26] Nishimura H Okazaki T Tanaka Y Nakatani K Hara M Matsumori A. Autoimmune dilated cardiomyopathy in PD-1 receptor-deficient mice. Science. (2001) 291:319–22. 10.1126/science.291.5502.319 11209085

[27] Robert C Schachter J Long GV Arance A Grob JJ Mortier L. Pembrolizumab versus ipilimumab in advanced melanoma. N Engl J Med. (2015) 372:2521–32. 10.1056/NEJMoa1503093 25891173

[28] Hamid O Robert C Daud A Hodi FS Hwu WJ Kefford R. Safety and tumor responses with lambrolizumab (anti-PD-1) in melanoma. N Engl J Med. (2013) 369:134–44. 10.1056/NEJMoa1305133 23724846 PMC4126516

[29] Baxi S Yang A Gennarelli RL Khan N Wang Z Boyce L. Immune-related adverse events for anti-PD-1 and anti-PD-L1 drugs: systematic review and meta-analysis. BMJ. (2018) 360:k793. 10.1136/bmj.k793 29540345 PMC5851471

[30] Monney L Sabatos CA Gaglia JL Ryu A Waldner H Chernova T. Th1-specific cell surface protein Tim-3 regulates macrophage activation and severity of an autoimmune disease. Nature. (2002) 415:536–41. 10.1038/415536a 11823861

[31] Zhu C Anderson AC Schubart A Xiong H Imitola J Khoury SJ. The Tim-3 ligand galectin-9 negatively regulates T helper type 1 immunity. Nat Immunol. (2005) 6:1245–52. 10.1038/ni1271 16286920

[32] Freeman GJ Casasnovas JM Umetsu DT DeKruyff RH. TIM genes: a family of cell surface phosphatidylserine receptors that regulate innate and adaptive immunity. Immunol Rev. (2010) 235:172–89. 10.1111/j.0105-2896.2010.00903.x 20536563 PMC2914464

[33] DeKruyff RH Bu X Ballesteros A Santiago C Chim YL Lee HH. T cell/transmembrane, Ig, and mucin-3 allelic variants differentially recognize phosphatidylserine and mediate phagocytosis of apoptotic cells. J Immunol. (2010) 184:1918–30. 10.4049/jimmunol.0903059 20083673 PMC3128800

[34] Huang YH Zhu C Kondo Y Anderson AC Gandhi A Russell A. CEACAM1 regulates TIM-3-mediated tolerance and exhaustion. Nature. (2015) 517:386–90. 10.1038/nature13848 25363763 PMC4297519

[35] Huang YH Zhu C Kondo Y Anderson AC Gandhi A Russell A. Corrigendum: CEACAM1 regulates TIM-3-mediated tolerance and exhaustion. Nature. (2016) 536:359. 10.1038/nature17421 26982724 PMC5110397

[36] Tang D Lotze MT. Tumor immunity times out: TIM-3 and HMGB1. Nat Immunol. (2012) 13:808–10. 10.1038/ni.2396 22910384 PMC3672065

[37] Gothert JR Eisele L Klein-Hitpass L Weber S Zesewitz ML Sellmann L. Expanded CD8+ T cells of murine and human CLL are driven into a senescent KLRG1+ effector memory phenotype. Cancer Immunol Immunother. (2013) 62:1697–709. 10.1007/s00262-013-1473-z 24022692 PMC11029347

[38] Grundemann C Schwartzkopff S Koschella M Schweier O Peters C Voehringer D. The NK receptor KLRG1 is dispensable for virus-induced NK and CD8+ T-cell differentiation and function *in vivo*. Eur J Immunol. (2010) 40:1303–14. 10.1002/eji.200939771 20201037

[39] Hofmann M Schweier O Pircher H. Different inhibitory capacities of human and mouse KLRG1 are linked to distinct disulfide-mediated oligomerizations. Eur J Immunol. (2012) 42:2484–90. 10.1002/eji.201142357 22684915

[40] de Jong R Stokkers P Lamme E Kool JM Borst F Brouwer M. Regulation of T-cell differentiation by CD2 and CD28 accessory molecules. Immunology. (1991) 74:175–82. 1684171 PMC1384590

[41] Hombach A Tillmann T Jensen M Heuser C Sircar R Diehl V. Specific activation of resting T cells against tumour cells by bispecific antibodies and CD28-mediated costimulation is accompanied by Th1 differentiation and recruitment of MHC-independent cytotoxicity. Clin Exp Immunol. (1997) 108:352–7. 10.1046/j.1365-2249.1997.3481245.x 9158110 PMC1904656

[42] Henson SM Akbar AN. KLRG1–more than a marker for T cell senescence. Age. (2009) 31:285–91. 10.1007/s11357-009-9100-9 19479342 PMC2813054

[43] Henson SM Franzese O Macaulay R Libri V Azevedo RI Kiani-Alikhan S. KLRG1 signaling induces defective Akt (ser473) phosphorylation and proliferative dysfunction of highly differentiated CD8+ T cells. Blood. (2009) 113:6619–28. 10.1182/blood-2009-01-199588 19406987

[44] Kaye J. CD160 and BTLA: LIGHTs out for CD4+ T cells. Nat Immunol. (2008) 9:122–4. 10.1038/ni0208-122 18204424

[45] Murphy KM Nelson CA Sedy JR. Balancing co-stimulation and inhibition with BTLA and HVEM. Nat Rev Immunol. (2006) 6:671–81. 10.1038/nri1917 16932752

[46] Freeman GCGJ. The CD160, BTLA, LIGHT/HVEM pathway: a bidirectional switch regulating T-cell activation. Immunol Rev. (2009) 229:244–58. 10.1111/j.1600-065X.2009.00783.x 19426226

[47] Chuang SS Kumaresan PR Mathew PA. 2B4 (CD244)-mediated activation of cytotoxicity and IFN-gamma release in human NK cells involves distinct pathways. J Immunol. (2001) 167:6210–6. 10.4049/jimmunol.167.11.6210 11714782

[48] Aldy KN Horton NC Mathew PA Mathew SO. 2B4+ CD8+ T cells play an inhibitory role against constrained HIV epitopes. Biochem Biophys Res Commun. (2011) 405:503–7. 10.1016/j.bbrc.2011.01.062 21256826 PMC3063451

[49] Bottino C Castriconi R Pende D Rivera P Nanni M Carnemolla B. Identification of PVR (CD155) and nectin-2 (CD112) as cell surface ligands for the human DNAM-1 (CD226) activating molecule. J Exp Med. (2003) 198:557–67. 10.1084/jem.20030788 12913096 PMC2194180

[50] Chan CJ Martinet L Gilfillan S Souza-Fonseca-Guimaraes F Chow MT Town L. The receptors CD96 and CD226 oppose each other in the regulation of natural killer cell functions. Nat Immunol. (2014) 15:431–8. 10.1038/ni.2850 24658051

[51] Chacon JA Schutsky K Powell DJ. The impact of chemotherapy, radiation and epigenetic modifiers in cancer cell expression of immune inhibitory and stimulatory molecules and anti-tumor efficacy. Vaccines. (2016) 4:43. 10.3390/vaccines4040043 27854240 PMC5192363

[52] Egelston CA Avalos C Tu TY Simons DL Jimenez G Jung JY. Human breast tumor-infiltrating CD8(+) T cells retain polyfunctionality despite PD-1 expression. Nat Commun. (2018) 9:4297. 10.1038/s41467-018-06653-9 30327458 PMC6191461

[53] Xie J Wang J Cheng S Zheng L Ji F Yang L. Expression of immune checkpoints in T cells of esophageal cancer patients. Oncotarget. (2016) 7:63669–78. 10.18632/oncotarget.11611 27577071 PMC5325394

[54] Zhou G Sprengers D Boor PPC Doukas M Schutz H Mancham S. Antibodies against immune checkpoint molecules restore functions of tumor-infiltrating T cells in hepatocellular carcinomas. Gastroenterology. (2017) 153:1107–19 e10. 10.1053/j.gastro.2017.06.017 28648905

[55] Tassi E Grazia G Vegetti C Bersani I Bertolini G Molla A. Early effector T lymphocytes coexpress multiple inhibitory receptors in primary non-small cell lung cancer. Cancer Res. (2017) 77:851–61. 10.1158/0008-5472.CAN-16-1387 27979840

[56] Fourcade J Sun Z Pagliano O Guillaume P Luescher IF Sander C. CD8(+) T cells specific for tumor antigens can be rendered dysfunctional by the tumor microenvironment through upregulation of the inhibitory receptors BTLA and PD-1. Cancer Res. (2012) 72:887–96. 10.1158/0008-5472.CAN-11-2637 22205715 PMC3288235

[57] Corrigan-Curay J Kiem HP Baltimore D O'Reilly M Brentjens RJ Cooper L. T-cell immunotherapy: looking forward. Mol Ther. (2014) 22:1564–74. 10.1038/mt.2014.148 25186558 PMC4435492

[58] Mahoney KM Rennert PD Freeman GJ. Combination cancer immunotherapy and new immunomodulatory targets. Nat Rev Drug Discov. (2015) 14:561–84. 10.1038/nrd4591 26228759

[59] Topalian SL Hodi FS Brahmer JR Gettinger SN Smith DC McDermott DF. Safety, activity, and immune correlates of anti-PD-1 antibody in cancer. N Engl J Med. (2012) 366:2443–54. 10.1056/NEJMoa1200690 22658127 PMC3544539

[60] Baitsch L Legat A Barba L Fuertes Marraco SA Rivals JP Baumgaertner P. Extended co-expression of inhibitory receptors by human CD8 T-cells depending on differentiation, antigen-specificity and anatomical localization. PLoS ONE. (2012) 7:e30852. 10.1371/journal.pone.0030852 22347406 PMC3275569

[61] Herndler-Brandstetter D Ishigame H Shinnakasu R Plajer V Stecher C Zhao J. KLRG1(+) effector CD8(+) T cells lose KLRG1, differentiate into all memory T cell lineages, and convey enhanced protective immunity. Immunity. (2018) 48:716–29 e8. 10.1016/j.immuni.2018.03.015 29625895 PMC6465538

[62] Messmer B Eissmann P Stark S Watzl C. CD48 stimulation by 2B4 (CD244)-expressing targets activates human NK cells. J Immunol. (2006) 176:4646–50. 10.4049/jimmunol.176.8.4646 16585556

[63] Bengsch B Seigel B Ruhl M Timm J Kuntz M Blum HE. Coexpression of PD-1, 2B4, CD160 and KLRG1 on exhausted HCV-specific CD8+ T cells is linked to antigen recognition and T cell differentiation. PLoS Pathog. (2010) 6:e1000947. 10.1371/journal.ppat.1000947 20548953 PMC2883597

[64] Song Y Wang B Song R Hao Y Wang D Li Y. T-cell Immunoglobulin and ITIM domain contributes to CD8(+) T-cell immunosenescence. Aging Cell. (2018) 17:e12716. 10.1111/acel.12716 29349889 PMC5847879

[65] Fons P Chabot S Cartwright JE Lenfant F L'Faqihi F Giustiniani J. Soluble HLA-G1 inhibits angiogenesis through an apoptotic pathway and by direct binding to CD160 receptor expressed by endothelial cells. Blood. (2006) 108:2608–15. 10.1182/blood-2005-12-019919 16809620

[66] Linsley CTLA-4 is a second receptor for the B cell acitvation antigen B7. J Exp Med. (1991) 174:561 10.1084/jem.174.3.561 1714933 PMC2118936

[67] Kwiecien I Stelmaszczyk-Emmel A Polubiec-Kownacka M Dziedzic D Domagala-Kulawik J. Elevated regulatory T cells, surface and intracellular CTLA-4 expression and interleukin-17 in the lung cancer microenvironment in humans. Cancer Immunol Immunother. (2017) 66:161–70. 10.1007/s00262-016-1930-6 27866241 PMC5281670

[68] Chen L Han X. Anti-PD-1/PD-L1 therapy of human cancer: past, present, and future. J Clin Invest. (2015) 125:3384–91. 10.1172/JCI80011 26325035 PMC4588282

[69] Chauvin JM Pagliano O Fourcade J Sun Z Wang H Sander C. TIGIT and PD-1 impair tumor antigen-specific CD8(+) T cells in melanoma patients. J Clin Invest. (2015) 125:2046–58. 10.1172/JCI80445 25866972 PMC4463210

[70] Koyama S Akbay EA Li YY Herter-Sprie GS Buczkowski KA Richards WG. Adaptive resistance to therapeutic PD-1 blockade is associated with upregulation of alternative immune checkpoints. Nat Commun. (2016) 7:10501. 10.1038/ncomms10501 26883990 PMC4757784

[71] Zahavi DJ Weiner LM. Targeting multiple receptors to increase checkpoint blockade efficacy. Int J Mol Sci. (2019) 20:E158. 10.3390/ijms20010158 30621125 PMC6337574

[72] Thommen DS Koelzer VH Herzig P Roller A Trefny M Dimeloe S. A transcriptionally and functionally distinct PD-1(+) CD8(+) T cell pool with predictive potential in non-small-cell lung cancer treated with PD-1 blockade. Nat Med. (2018) 24:994–1004. 10.1038/s41591-018-0057-z 29892065 PMC6110381

[73] Kim H-D Song G-W Park S Jung MK Kim MH Kang HJ. Association between expression level of PD1 by tumor-infiltrating CD8+ T cells and features of hepatocellular carcinoma. Gastroenterology. (2018) 155:1936–50.e17. 10.1053/j.gastro.2018.08.030 30145359

