# Supplementary material for: A Comprehensive Analysis of Key Immune Checkpoint Receptors on Tumor-Infiltrating T Cells From Multiple Types of Cancer
Source: Front Oncol. 2019 Oct 25;9:1066. doi: 10.3389/fonc.2019.01066 (PMC6823747; doi:10.3389/fonc.2019.01066)
Supplement: Supplementary file 3 [file Data_Sheet_1.pdf]

Supplementary Figure 1:

CD8 | **CD4**

A:

R=0.634 P<0.0001****

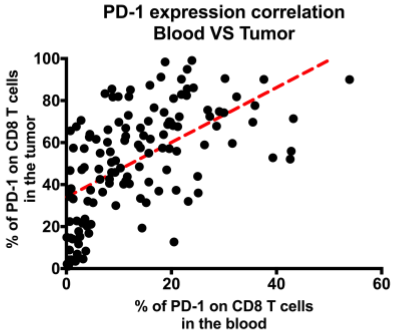

B:

R=0.800 P<0.0001****

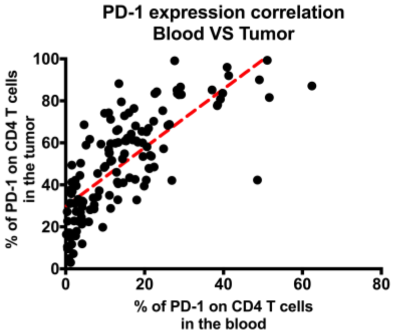

C:

R=0.436 P<0.0001****

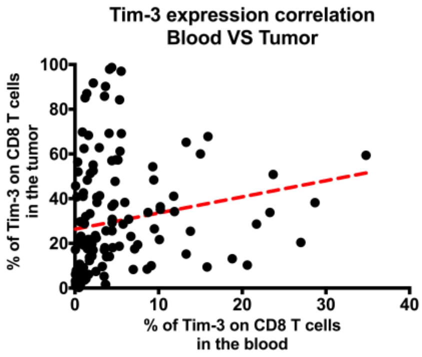

D:

R=0.535 P<0.0001****

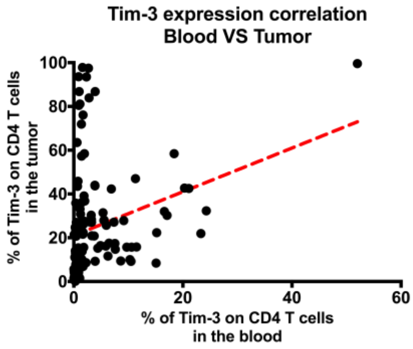

E:

R=0.243 P=0.055 **NS**

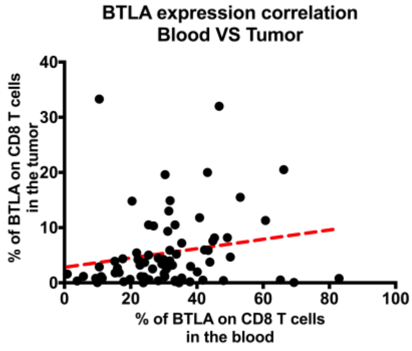

F:

R=0.0303 P=0.802 **NS**

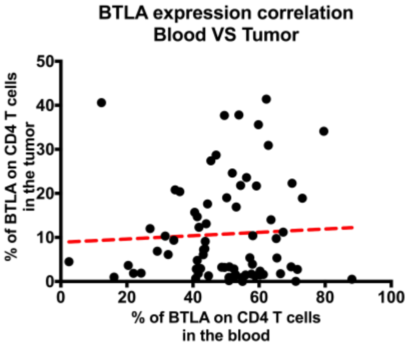

Supplementary Figure 2:

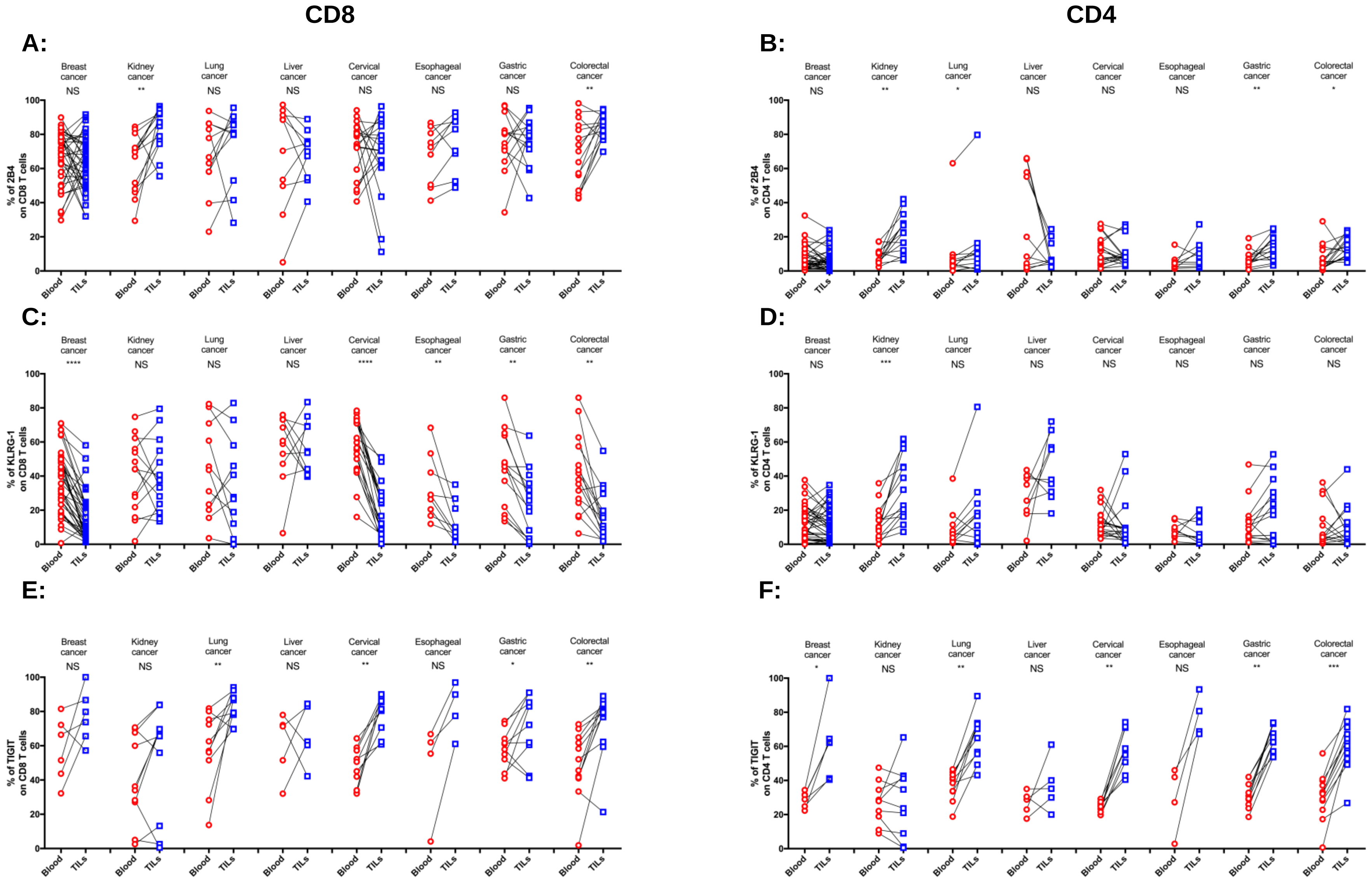

G:

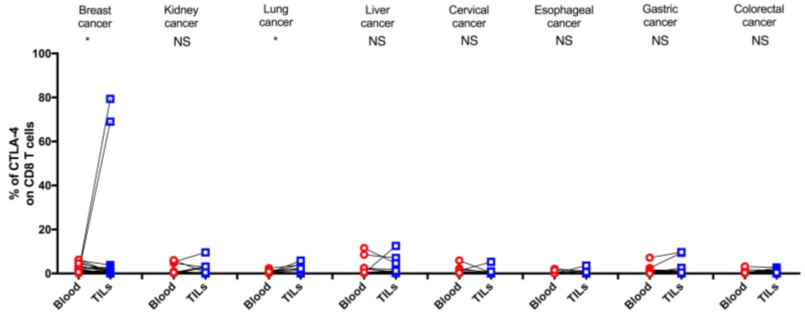
Breast cancer
*
Kidney cancer
NS
Lung cancer
*
Liver cancer
NS
Cervical cancer
NS
Esophageal cancer
NS
Gastric cancer
NS
Colorectal cancer
NS
% of CTLA-4 on CD8 T cells
0
20
40
60
80
100
Blood
TILs

H:

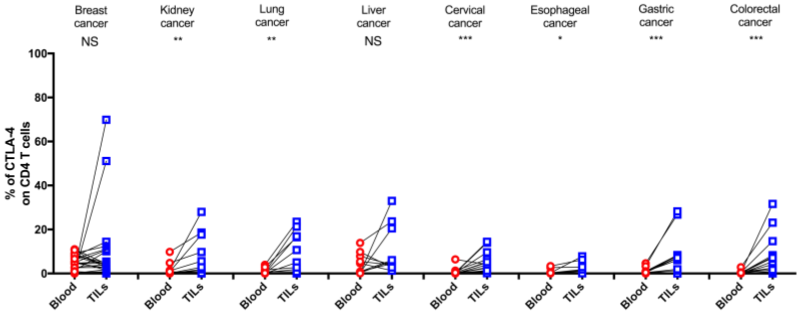
Breast cancer
NS
Kidney cancer
**
Lung cancer
**
Liver cancer
NS
Cervical cancer
***
Esophageal cancer
*
Gastric cancer
***
Colorectal cancer
***
% of CTLA-4 on CD4 T cells
0
20
40
60
80
100
Blood
TILs

I:

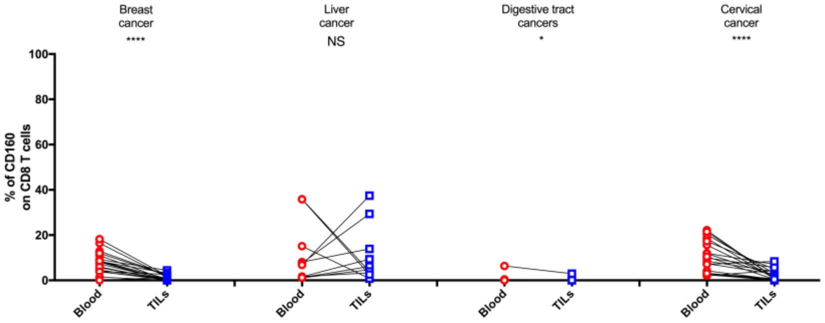
Breast cancer
****
Liver cancer
NS
Digestive tract cancers
*
Cervical cancer
****
% of CD160 on CD8 T cells
0
20
40
60
80
100
Blood
TILs

J:

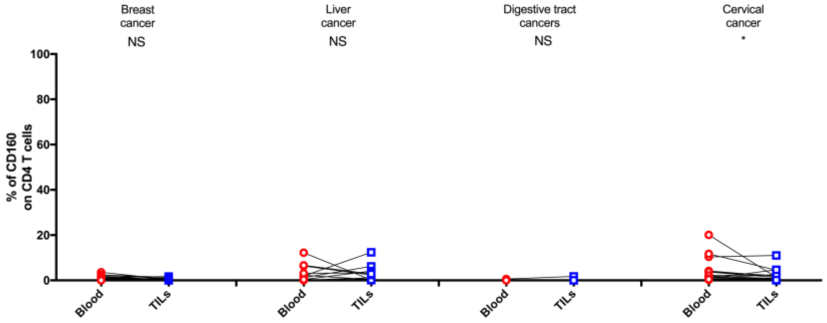
Breast cancer
NS
Liver cancer
NS
Digestive tract cancers
NS
Cervical cancer
*
% of CD160 on CD4 T cells
0
20
40
60
80
100
Blood
TILs

Supplementary Figure 3:

A:

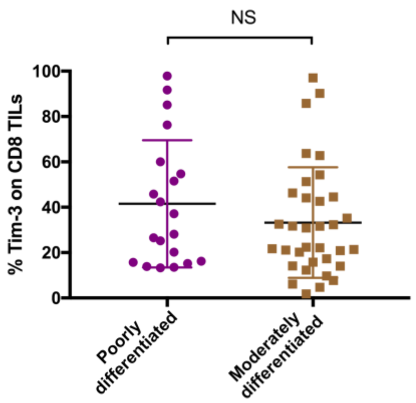

B:

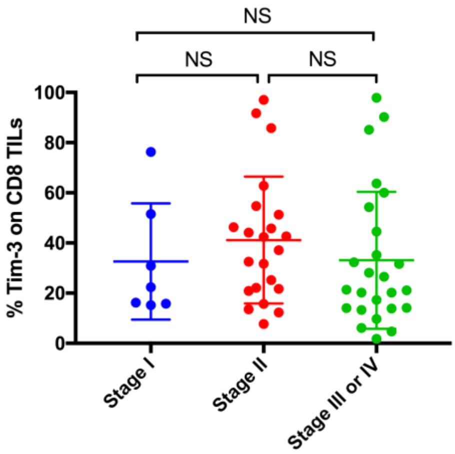

Supplementary Figure 4:
A:
8-color panel 1: Blood

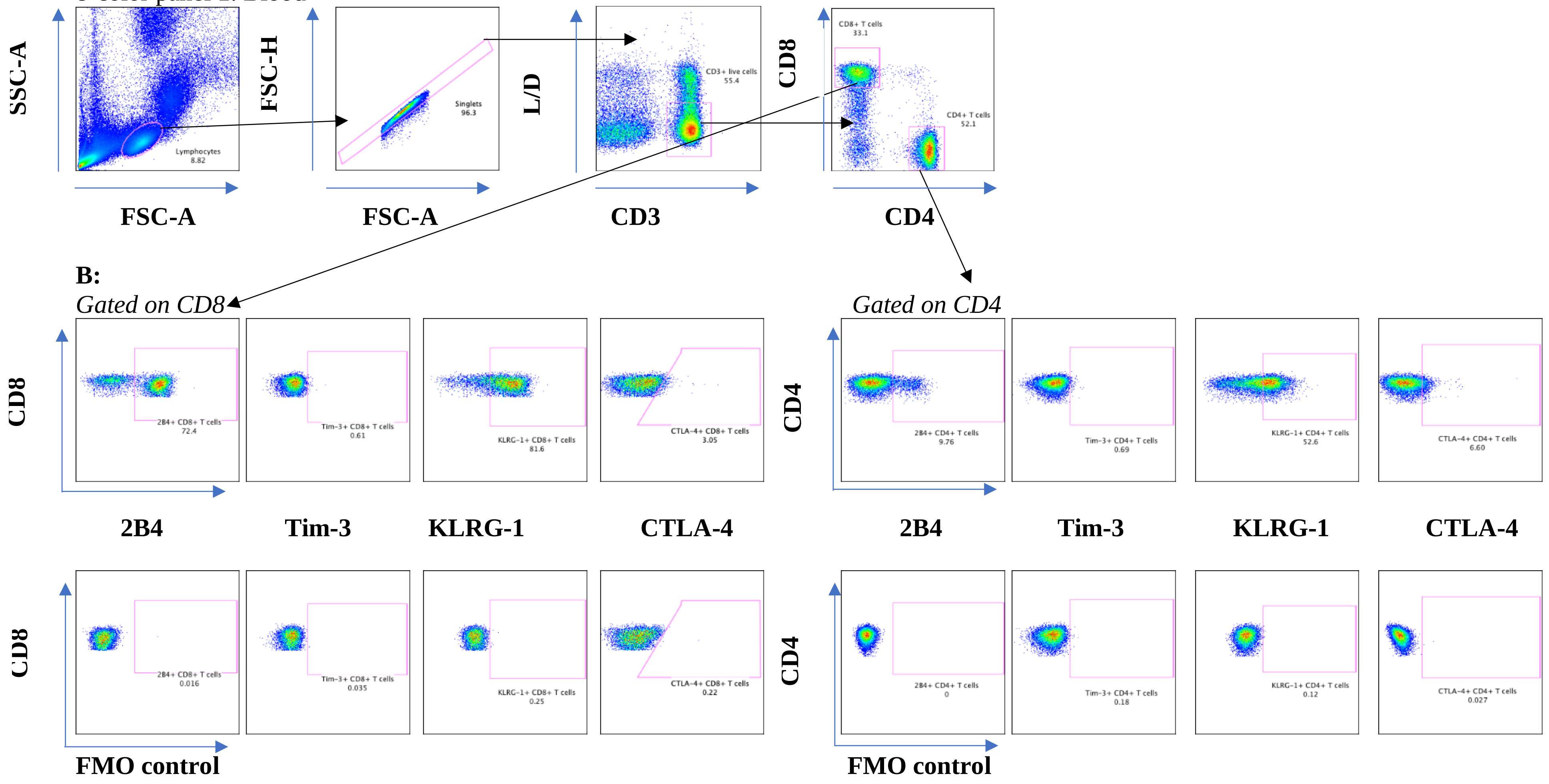

Supplementary Figure 5:
A:
8-color panel 1: Tumor

B:

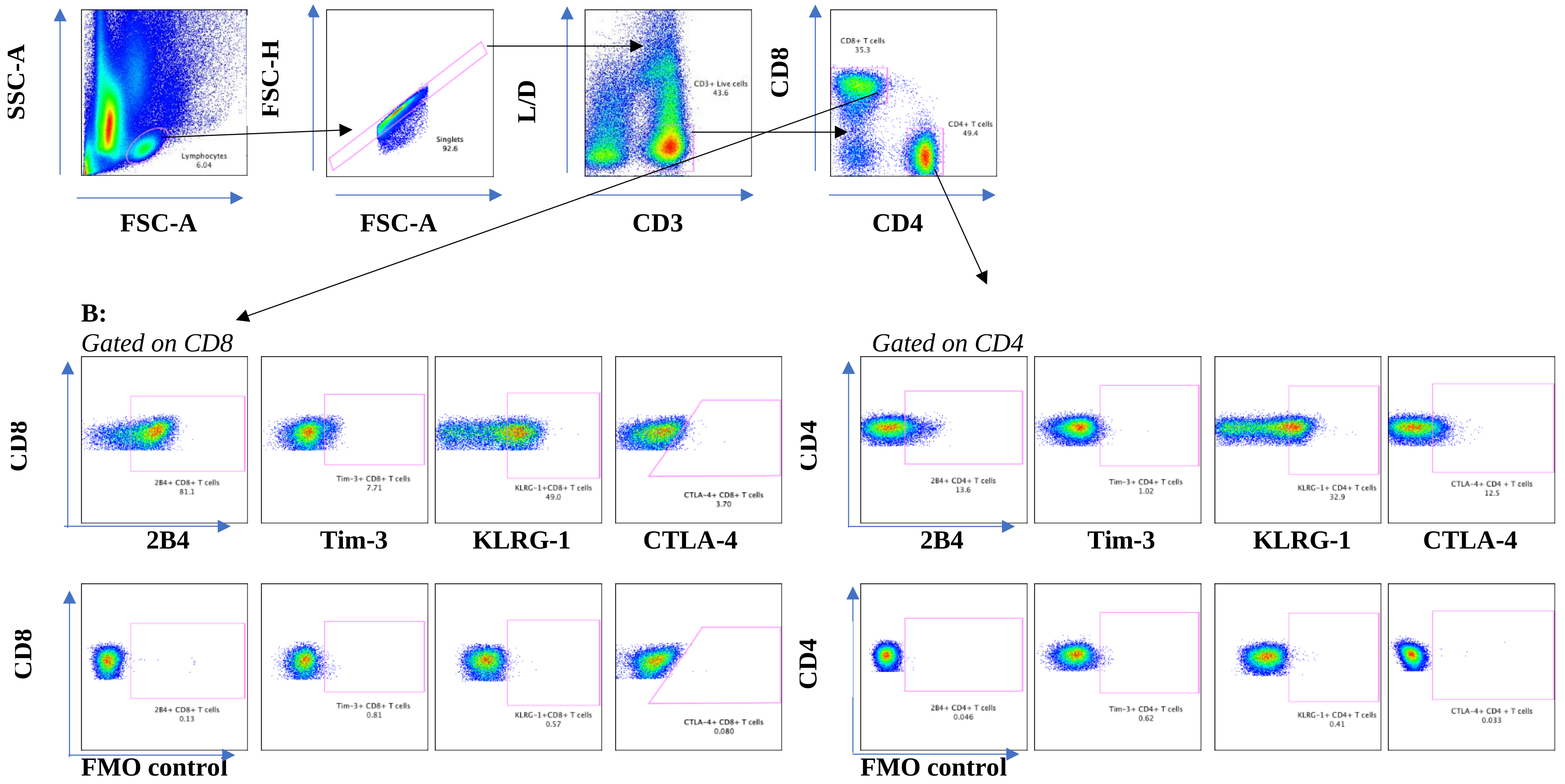

Supplementary Figure 6:

A:
8-color panel 2: Blood

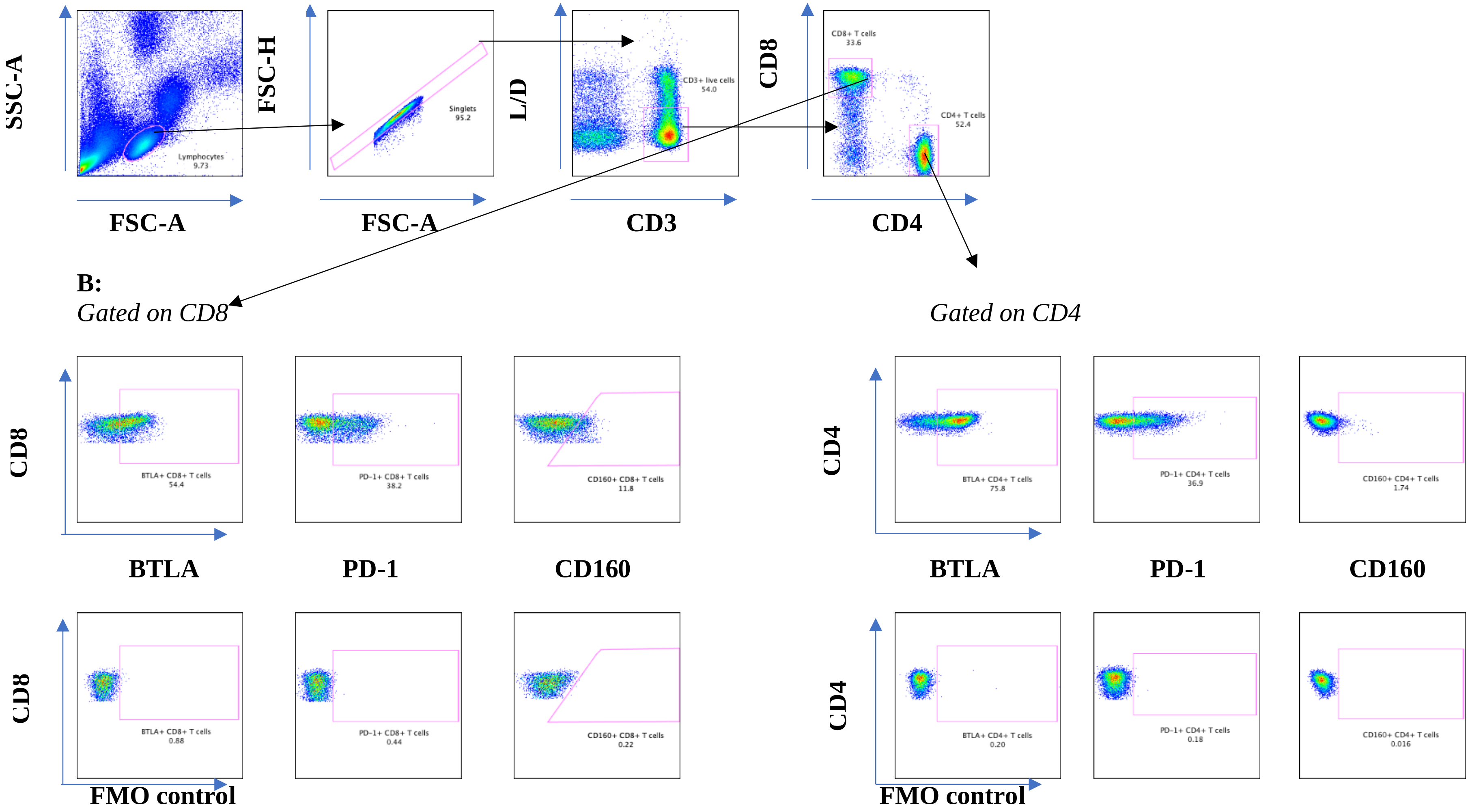

Supplementary Figure 7:

A:

8-color panel 2: Tumor

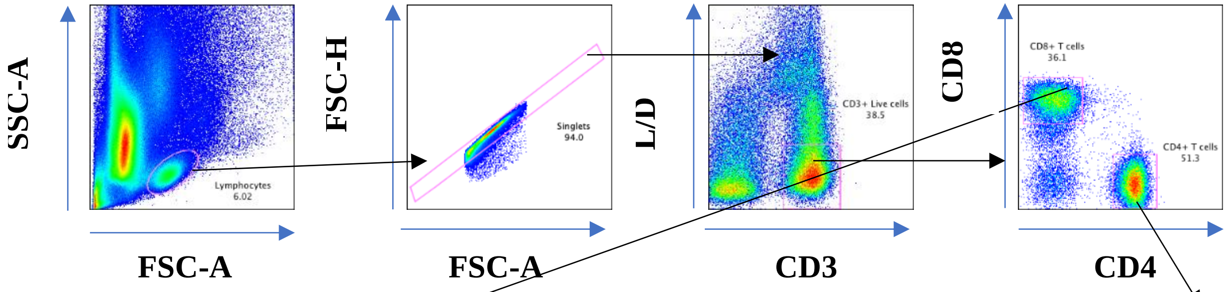

B:

Gated on CD8

Gated on CD4

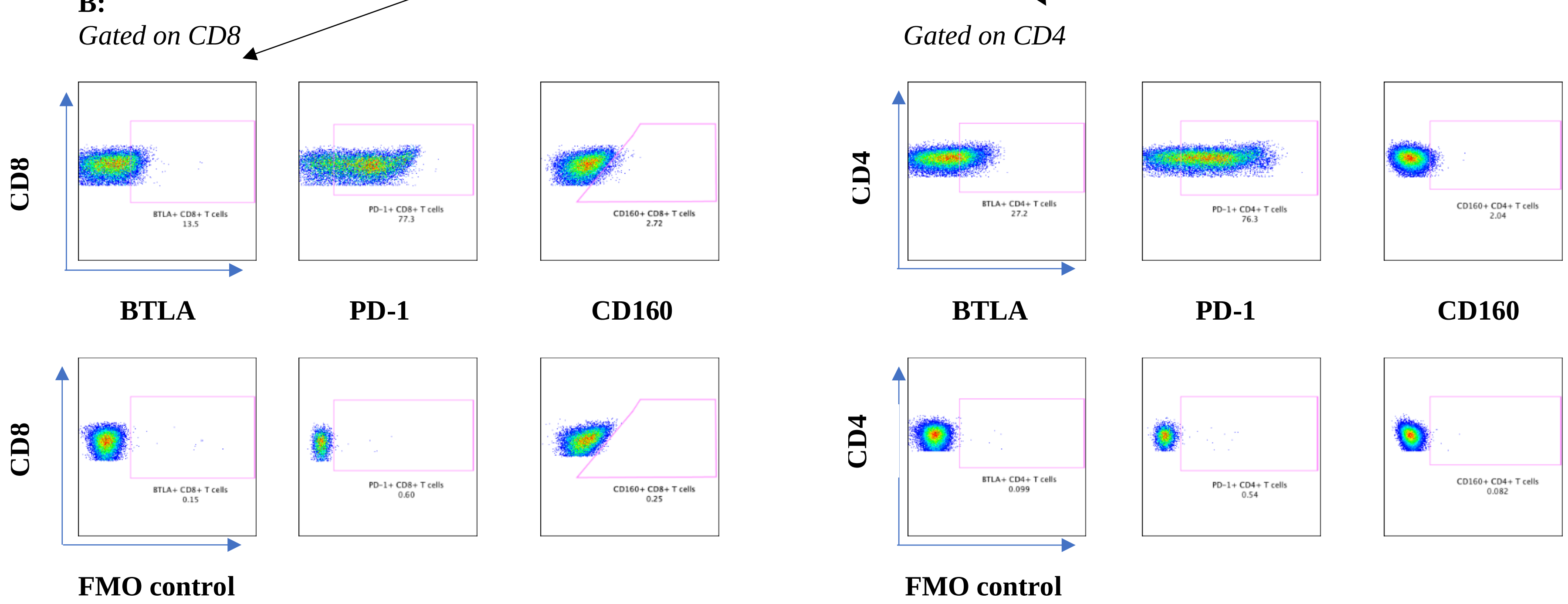

Supplementary Figure 8:

A:

14-color panel: Tumor

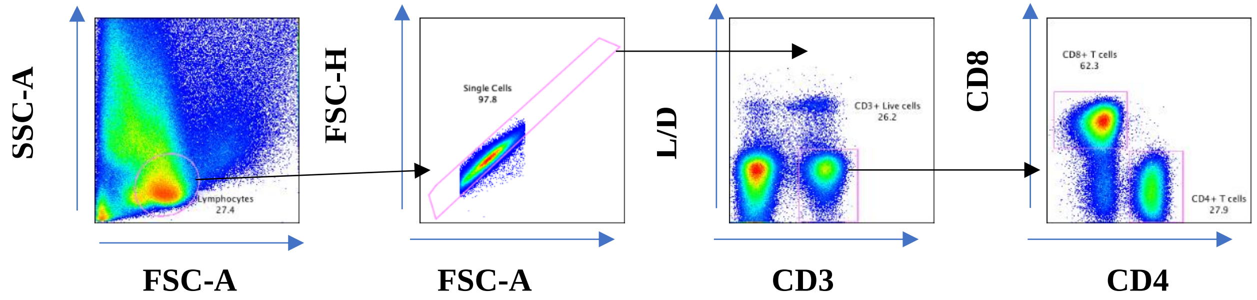

B:

Gated on CD8

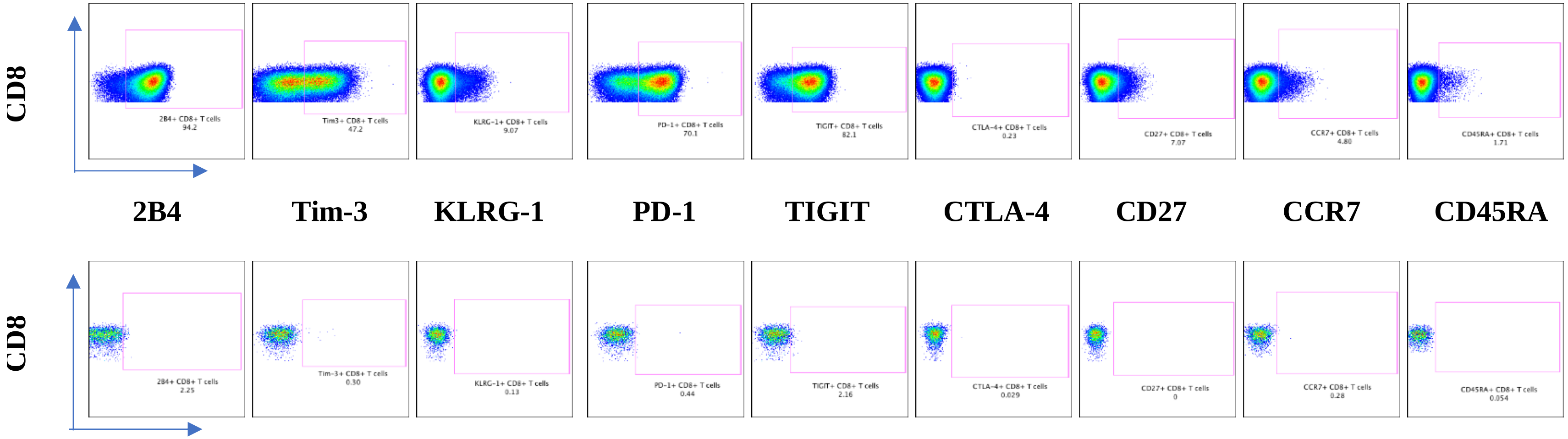

FMO control

C:

Gated on CD4

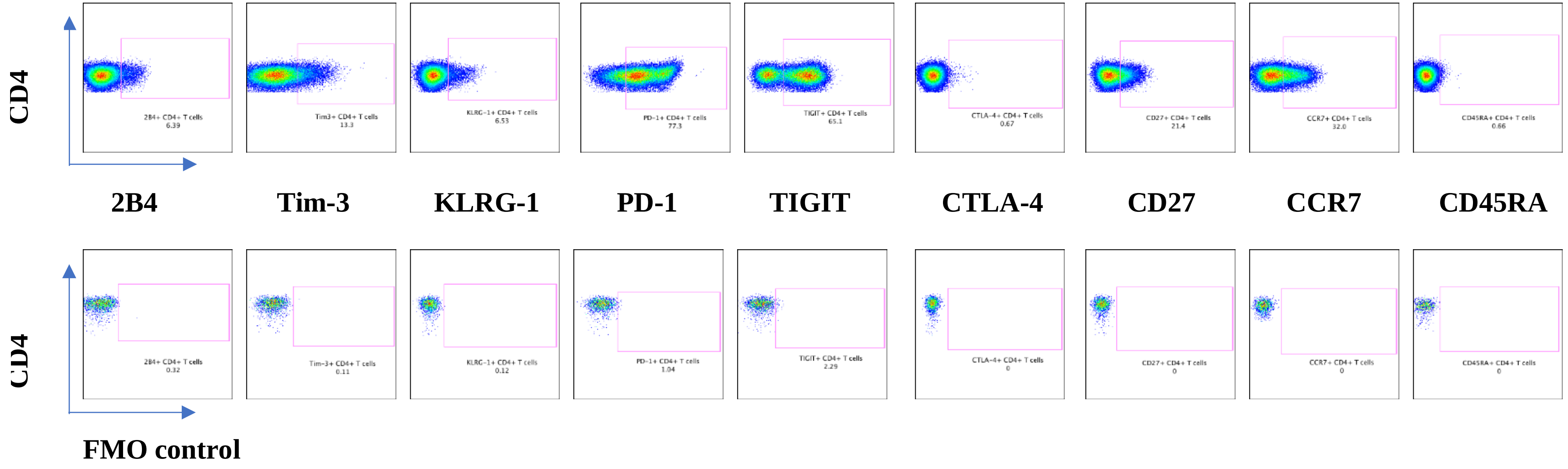

D:

Gated on CD8

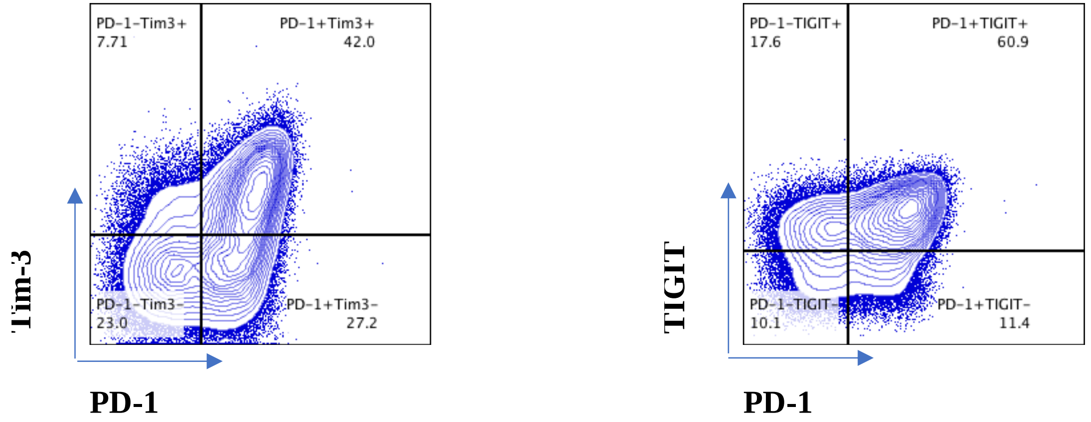

Supplementary Figure 1: PD-1+ and Tim-3+ but not BTLA+ frequency in peripheral T cells positively correlates with that on TILs

Correlation analysis of the frequency of PD-1+, Tim-3+ and BTLA+ cells in CD4 and CD8 T cell populations between peripheral blood and tumor from 131 (analyses of PD-1 and Tim-3) and 74 (analysis of BTLA) blood-tumor matched primary cancer patients respectively (see **Fig. 1** for details). P values and correlation coefficients (r) were calculated with Spearman's correlation test, ****P < 0.0001.

Supplementary Figure 2: Distinct surface expression of 2B4, CD160, TIGIT, KLRG-1 and CTLA-4 on T cells from peripheral blood vs tumor across eight types of cancer patients

FACS analysis of 2B4 (**A, B**), KLRG-1 (**C, D**) and CTLA-4 (**G, H**) on CD8 (**left**) and CD4 (**right**) T cells from 131 blood-tumor matched primary cancer patients (see Fig. 1 for details). FACS analysis of TIGIT on CD8 (**E**) and CD4 (**F**) T cells from 6 blood-tumor matched breast cancer patients, 10 kidney cancer patients, 10 lung cancer patients, 5 liver cancer patients, 10 cervical cancer patients, 4 oesophageal cancer patients, 10 gastric cancer patients and 12 colorectal cancer patients. CD160 on CD8 (**I**) and CD4 (**J**) T cells from 74 cancer patients (same cohort as that in the analysis of BTLA), with 32 blood-tumor matched breast cancer patients, 10 liver cancer patients, 12 digestive tract cancer patients (5 oesophageal cancer, 4 gastric cancer and 3 colorectal cancer) and 20 cervical cancer patients. Wilcoxon paired t test was performed to detect the statistical significance (*P<0.0332, **P<0.0021, ***P<0.0002, ****P<0.0001). Frequency in peripheral blood VS tumor is in (%).

Supplementary Figure 3: Tim-3 expression on CD8 TILs is not linked with cervical cancer stage

(**A**) FACS analysis of Tim-3 on CD8 TILs isolated from poorly differentiated and moderately differentiated cervical cancer samples. (**B**) FACS analysis of Tim-3 on CD8 TILs isolated from samples of different cervical cancer stages. In the association analysis of Tim-3 surface expression on CD8 TILs with cancer differentiation and cancer stages, Mann Witney t test was performed to detect the statistical significance (*P<0.0332, **P<0.0021, ***P<0.0002, ****P<0.0001).

Supplementary Figure 4-8: Gating strategy of IRs on CD4 and CD8 T cells

From 2012 to 2014, two 8-color panels were applied for *ex vivo* phenotypic analysis. 7 inhibitory receptors were designed into 2 panels and exemplary gating strategy plots of a blood-tumor matched breast cancer patient were shown in (**Supplementary Figure 4-5**). Progressive gating strategy was used to exclude doublets and dead cells and to identify CD4 and CD8 T cells afterwards (**A in Supplementary Figure 4-8**). FMO controls were applied accordingly in order to properly position gates of IRs (**Bottom rows of B in Supplementary Figure 4-8 and the bottom row of C in Supplementary Figure 8**). We did not apply FMO controls of live/dead staining, CD3, CD4 and CD8. FMO controls were initially conducted on weekly basis to position gates of IRs and then changed to biweekly basis. The cocktails of FMOs for all the panels are attached in **Supplementary Table 3** below. The number of cells in each FMO control (mainly in the study conducted by the 14-color panel) was different from the full staining sample. This is due to the fact that only limited number of viable TILs can be obtained from an individual patient and TILs isolated from the sample of the individual cancer patient had to be equally divided for the staining of multiple FMO controls. From 2014

onwards with an upgrade of the filters in flow cytometer, a 14-color panel was used for the surface analysis of 6 inhibitory receptors on T cells, which allowed us to investigate the co-expression of multiple IRs on TILs. Reviewing the results from the study in the first 2 years, we observed the low expression of CD160 and BTLA on TILs from cancer patients. Therefore, in this 14-color panel, we decided to exclude BTLA and CD160 from the updated panel due to their low expressions on TILs and add TIGIT (a newly identified IR at that time) and 3 T-cell differentiation markers (CD27, CCR7 and CD45RA) to the 14-color panel. Gating strategy of the markers in this 14-color panel on TILs from a cervical cancer patient was shown on **Supplementary Figure 8-A.** Representative Boolean gating strategy was shown on **Supplementary Figure 8-B.** In the co-expression analysis of multiple IRs on CD8 TILs**,** Simplified Presentation of Incredibly Complex Evaluations (SPICE) analysis was applied to investigate the co-expression of PD-1, Tim-3, 2B4, TIGIT, KLRG-1 and CTLA-4 on CD8 TILs from multiple cancer patients. With 6 IRs investigated, there are 64 possible combinations/subsets on CD8 TILs, which were calculated by the algorithms of Boolean combination gates in Flowjo software before transferring to the SPICE software. After identifying the shared subset of CD8 TILs, we further analysed the percentage of 5 T-cell differentiation stages in the shared subset of CD8 TILs from 10 cervical cancer patients by the Boolean combination gates. For the co-expression analysis between PD-1 and Tim-3 or between PD-1 and TIGIT, gating strategy was shown in **Supplementary Figure 8D**.

Supplementary Information about clinical cohorts

TILs from tumor tissue samples were freshly isolated right after the surgeries. In order to ensure the quality of FACS data, we ruled out any tumor samples in which

the viable CD3+ TILs were lower than 10,000 cells. From 2012 to 2014, as mentioned above, two 8-color FACS panels were used for the analysis of IRs on T cells. Therefore, some of the patients in the cohort only have the valid data from 1 panel after the quality control check due to the limited number of TILs yielded from the surgical samples. That is also the reason why in each figure in the manuscript, the number of patients varies. All the patients (74 patients) from multiple types of cancer in the analyses of BTLA and CD160 are identical since both markers are designed in the same panel. All the patients for the analysis of TIGIT (**Supplementary Figure 2E**) are included in the co-expression studies (**Figure 3 and 4**). However, co-expression studies in **Figure 3 and 4** contain additional patients in whom we received tissue samples only rather than blood-tissue paired samples. All the details of the patients in each cohort are listed in **Supplementary Table 4** below and marked in the clinical characteristics of patients in **Supplementary Table 1**.

Supplementary Information about cancer differentiation

Cancer differentiation grade of tumor samples is determined by pathologists under microscopic examination and has 3 grades; well-differentiation, poor-differentiation and moderately-differentiation. The principles are listed below.

Well-differentiated carcinomas have relatively normal-looking cells that do not appear to be growing rapidly. These cancers tend to grow and spread slowly and have a better prognosis.

Poorly differentiated carcinomas lack normal features, tend to grow and spread faster, and have a worse prognosis.

Moderately differentiated carcinomas have features and a prognosis in between these two.

Supplementary Table 3:

A: FMO controls for 8-color panel 1

Fluorophore	CTLA-4 FMO	Tim3 FMO	KLRG-1 FMO	2B4 FMO
**PE-Cy7**	---	CTLA-4	CTLA-4	CTLA-4
**BV421**	Tim-3	---	Tim-3	Tim-3
**BV605**	KLRG-1	KLRG-1	---	KLRG-1
**APC**	2B4	2B4	2B4	---
**BV650**	PD-1	PD-1	PD-1	PD-1
**AF 700**	CD3	CD3	CD3	CD3
**FITC**	CD4	CD4	CD4	CD4
**APC-H7**	CD8	CD8	CD8	CD8
**BV510**	L/D Aqua	L/D Aqua	L/D Aqua	L/D Aqua

B: FMO controls for 8-color panel 2

Fluorophore	CD160 FMO	BTLA FMO	PD-1 FMO
**PE-Cy7**	---	CD160	CD160
**APC**	BTLA	---	BTLA
**BV650**	PD-1	PD-1	---
**AF 700**	CD3	CD3	CD3
**FITC**	CD4	CD4	CD4
**APC-H7**	CD8	CD8	CD8
**BV510**	L/D Aqua	L/D Aqua	L/D Aqua

C: FMO controls for 14-coloar panel

Fluorophore	CTLA-4 FMO	2B4 FMO	PD-1 FMO	TIGIT FMO	CD27 FMO	CD45RA FMO	CCR7 FMO	CD56 FMO
**PE-Cy7**	---	CTLA-4	CTLA-4	CTLA-4	CTLA-4	CTLA-4	CTLA-4	CTLA-4
**BV421**	Tim-3	Tim-3	Tim-3	Tim-3	Tim-3	Tim-3	Tim-3	Tim-3
**BV605**	KLRG-1	KLRG-1	KLRG-1	KLRG-1	KLRG-1	KLRG-1	KLRG-1	KLRG-1
**APC**	2B4	---	2B4	2B4	2B4	2B4	2B4	2B4
**BV650**	PD-1	PD-1	---	PD-1	PD-1	PD-1	PD-1	PD-1
**PE**	TIGIT	TIGIT	TIGIT	---	TIGIT	TIGIT	TIGIT	TIGIT
**PERCP5.5**	CD27	CD27	CD27	CD27	---	CD27	CD27	CD27
**BV785**	CD45RA	CD45RA	CD45RA	CD45RA	CD45RA	---	CD45RA	CD45RA
**BV711**	CCR7	CCR7	CCR7	CCR7	CCR7	CCR7	---	CCR7
**PE-Dazzle594**	CD56	CD56	CD56	CD56	CD56	CD56	CD56	---
**AF 700**	CD3	CD3	CD3	CD3	CD3	CD3	CD3	CD3
**FITC**	CD4	CD4	CD4	CD4	CD4	CD4	CD4	CD4
**APC-H7**	CD8	CD8	CD8	CD8	CD8	CD8	CD8	CD8
**BV510**	L/D Aqua	L/D Aqua	L/D Aqua	L/D Aqua	L/D Aqua	L/D Aqua	L/D Aqua	L/D Aqua

Supplementary Table 4:

Cohorts	Total number of patients recruited in this study	No. of patients in the analyses of PD-1, Tim-3, KLRG-1 and 2B4 in Figure 1 and 2	No. of patients in the analyses of CD160 and BTLA / No. of patients appearing in the analyses of PD-1, Tim-3, KLRG-1 and 2B4 in Figure 1 and 2	No. of patients in the analysis of TIGIT / No. of patients appearing in the analyses of PD-1, Tim-3, KLRG-1 and 2B4 in Figure 1 and 2	No. of patients in Figure 3 and 4 / No. of patients appearing in the analyses of PD-1, Tim-3, KLRG-1 and 2B4 in Figure 1 and 2
**Breast cancer cohort**	39	38	32 / 32	6 / 6	7 / 6
**Liver cancer cohort**	15	10	10 / 5	5 / 5	5 / 5
**Lung cancer cohort**	11	11	0 / 0	10 / 10	10 /10
**Oesophageal cancer cohort**	9	9	5 / 5	4 / 4	4 / 4
**Gastric cancer cohort**	15	14	4 /4	10 / 10	11 / 10
**Colorectal cancer cohort**	15	15	3 / 3	12 / 12	12 /12
**Kidney cancer cohort**	14	14	0 / 0	10 / 10	10 / 10
**Cervical cancer cohort**	54	20	20 / 20	10 / 10	10 /10
